# TREM2 modulates differential deposition of modified and non-modified Aβ species in extracellular plaques and intraneuronal deposits

**DOI:** 10.1186/s40478-021-01263-x

**Published:** 2021-10-18

**Authors:** Pranav Joshi, Florian Riffel, Sathish Kumar, Nàdia Villacampa, Sandra Theil, Samira Parhizkar, Christian Haass, Marco Colonna, Michael T. Heneka, Thomas Arzberger, Jochen Herms, Jochen Walter

**Affiliations:** 1 Department of Neurology, University of Bonn, Venusberg-Campus 1, (Formerly Sigmund-Freud-Str. 25), 53127 Bonn, Germany; 2 Department of Neurodegenerative Diseases and Gerontopsychiatry, University Hospital Bonn, Bonn, Germany; 3 Neuroinflammation Unit, German Center for Neurodegenerative Diseases e. V. (DZNE), Bonn, Germany; 4 Biomedical Center (BMC), Faculty of Medicine, Ludwig-Maximilians-Universität München, Munich, Germany; 5 Munich Cluster for Systems Neurology (SyNergy), Munich, Germany; 6 Molecular Neurodegeneration Unit, German Center for Neurodegenerative Diseases e.V. (DZNE) Munich, Munich, Germany; 7 Department of Pathology and Immunology, Washington University School of Medicine, St. Louis, USA; 8 Center for Neuropathology and Prion Research, Ludwig-Maximilians-Universität München, Munich, Germany; 9 Department of Psychiatry and Psychotherapy, Ludwig-Maximilians-Universität München, Munich, Germany

**Keywords:** TREM2, Microglia, Post-translational modification, Aβ, Intraneuronal, Vascular deposits

## Abstract

Progressive accumulation of Amyloid-β (Aβ) deposits in the brain is a characteristic neuropathological hallmark of Alzheimer’s disease (AD). During disease progression, extracellular Aβ plaques undergo specific changes in their composition by the sequential deposition of different modified Aβ species. Microglia are implicated in the restriction of amyloid deposits and play a major role in internalization and degradation of Aβ. Recent studies showed that rare variants of the Triggering Receptor Expressed on Myeloid cells 2 (TREM2) are associated with an increased risk for AD. Post-translational modifications of Aβ could modulate the interaction with TREM2, and the uptake by microglia. Here, we demonstrate that genetic deletion of TREM2 or expression of a disease associated TREM2 variant in mice lead to differential accumulation of modified and non-modified Aβ species in extracellular plaques and intraneuronal deposits. Human brains with rare TREM2 AD risk variants also showed altered deposition of modified Aβ species in the different brain lesions as compared to cases with the common variant of TREM2. These findings indicate that TREM2 plays a critical role in the development and the composition of Aβ deposits, not only in extracellular plaques, but also intraneuronally, that both could contribute to the pathogenesis of AD.

**Supplementary Information:**

The online version contains supplementary material available at 10.1186/s40478-021-01263-x.

## Background

Alzheimer’s disease (AD) is characterized neuropathologically by the combined occurrence of extracellular amyloid-beta (Aβ) plaques and intracellular neurofibrillary tangles (NFTs) with abnormally phosphorylated tau (τ) protein in the brain [24, 65]. Aβ deposits in the human brain contain different Aβ species, including N‐terminal truncated, pyroglutamated, phosphorylated and nitrated variants that show significant differences in aggregation, stability, and toxicity [1, 42]. In particular, Aβ peptides with pyroglutamate-modification at glutamate residue 3 (N3pE-Aβ) or phosphorylated serine residue 8 (pSer8-Aβ) have increased propensity to form aggregates with increased neurotoxicity [38, 40–42, 53]. Previous investigations revealed a specific sequential deposition starting with non-modified Aβ (nmAβ) peptides, followed by N3pE-Aβ and pSer8-Aβ species in extracellular plaques during the progression from pre-clinical to clinical phases of AD [1, 54]. A similar sequence for the deposition of these Aβ species was also found in cerebral amyloid angiopathy (CAA) [11].

Genome-wide association studies (GWAS) and exome sequencing have revealed genetic loci related to inflammatory pathways to be associated with an increased risk for AD [5, 18, 71]. Among these subsets of genes, rare variants of the microglial transmembrane receptor, Triggering Receptor Expressed on Myeloid cells (TREM2), confer a high risk for the development of AD, comparable to the risk exerted by the Apolipoprotein E4 allele (ApoE4) [18, 30]. TREM2 is preferentially expressed in microglia and functions as a receptor for different ligands, including anionic lipids, ApoE, and Aβ [12, 47, 74, 82]. Activation of TREM2 regulates microglial functions, including phagocytosis, cytokine production, proliferation and migration [16, 69, 75]. TREM2 is proteolytically processed by ADAM proteases to generate soluble variants of TREM2 (sTREM2) [25, 34, 78], that can be detected in extracellular fluids. sTREM2 could act as a decoy receptor to negatively modulate TREM2 signaling and inflammatory responses of microglia, and also shows trophic activity to promote microglial survival [35, 83].

TREM2 positive microglia cluster around extracellular plaques in brains of human AD cases and amyloid precursor protein (APP) transgenic mice, and the deletion of TREM2 in APP mouse models  results in  altered morphology and seeding of plaques, as well as decrease in number of plaque associated microglia [28, 51, 67], indicating an involvement of TREM2 in the restriction of Aβ deposits [11, 75]. Here, we sought to characterize the role of TREM2 in accumulation and distribution of modified and non-modified Aβ species in the brain. Deletion of TREM2 or the expression of the disease associated TREM2^T66M^ variant in different APP transgenic mouse models led to altered composition not only of extracellular plaques, but also of intraneuronal deposits containing modified and non-modified Aβ variants. Human cases with rare AD associated TREM2 variants also showed altered composition and morphology of the different Aβ pathological lesions as compared to AD cases with the common TREM2 variant. Together, the data indicate an important role of TREM2 in altering the composition of Aβ related brain lesions during the pathogenesis of AD.

## Methods

### Transgenic mice

APP695KM670/671NL; PS1L166P TREM2^+/+^ and APP695KM670/671NL; PS1L166P TREM2^−/−^ transgenic mice, and 5xFAD TREM2^+/+^ and 5xFAD TREM2^−/−^ transgenic mice were described previously [31, 51, 66, 75].

12 M old female APPKM670/671NL; PS1ΔE9 transgenic mice endogenously expressing TREM2 WT or the homozygous TREM2^T66M^ knock‐in (KI) mutation were obtained from Taconic Biosciences GmbH, Cologne, Germany [31]. The different mouse models are described in Table 1.

**Table 1:** Details of transgenic mouse models and quantification of Aβ plaque pathology. n.d.-not detected, ROI-Region of interest

Sr. No.	Transgenic mouse models	Age (months)	Sex	Genotype	No. of animals (n)		Total number of Aβ plaques/ROI stained with various antibodies														
							Somatosensory cortex (SSC)					Retrosplenial cortex (RSC)					Dentate gyrus (DG)				
							pSer8-Aβ	N3p3-Aβ	nmAβ	Aβ (4G8)	Aβ (2964)	pSer8-Aβ	N3p3-Aβ	nmAβ	Aβ (4G8)	Aβ (2964)	pSer8-Aβ	N3p3-Aβ	nmAβ	Aβ (4G8)	Aβ (2964)
1	5xFAD	5 M	Male	TREM2^+/+^	5	Total plaque count/group	1117	1664	1791	1847	1842	1353	1770	1724	1777	1815	933	1263	1932	1872	1857
						Plaque load (%)	2.89	8.11	10.91	11.42	11.46	4.35	7.49	7.71	9.27	8.52	2.89	5.12	8.76	8.98	8.93
				TREM2^−/−^	5	Total plaque count/group	1631	1836	2247	2314	2305	1734	1899	2120	2169	2198	1257	1403	2258	2196	2183
						Plaque load (%)	9.53	10.80	17.90	19.08	17.49	7.66	9.29	13.82	14.41	14.45	5.27	6.94	13.55	14.29	14.22
		15 M	Male	TREM2^+/+^	5	Total plaque count/group	1565	2322	3616	3725	3833	2057	2559	3641	3802	3778	1530	2535	3426	3537	3461
						Plaque load (%)	4.59	17.26	27.31	28.10	29.38	7.09	13.19	25.04	25.84	26.41	5.49	10.24	21.40	21.51	21.00
				TREM2^−/−^	5	Total plaque count/group	2250	3013	4414	4417	4523	2846	3285	4398	4520	4488	2153	3139	4033	4144	4096
						Plaque load (%)	13.06	23.76	41.46	41.37	42.44	15.73	21.93	32.66	35.63	33.95	10.99	16.07	32.16	32.39	31.95
2	APP/PS1L166P	4 M	Male	TREM2^+/+^	5	Total plaque count/group	422	n.d.﻿	1027	967	982	420	n.d.﻿	852	892	917	245	n.d.﻿	626	760	776
						Plaque load (%)	1.93	n.d.	5.51	4.96	5.25	2.46	n.d.﻿	5.59	6.52	6.62	1.35	n.d.﻿	4.14	5.03	4.63
				TREM2^−/−^	5	Total plaque count/group	607	n.d.﻿	1215	1156	1193	631	n.d.﻿	1062	1115	1132	446	n.d.	845	957	988
						Plaque load (%)	2.93	n.d.﻿	8.19	9.03	9.88	4.09	n.d.﻿	7.64	9.14	9.18	2.46	n.﻿d.	6.57	7.01	7.63
		12 M	Female	TREM2^+/+^	5	Total plaque count/group	804	1935	4003	4090	3959	942	2096	3776	4050	3943	649	1584	2437	2482	2485
						Plaque load (%)	4.81	14.99	28.03	27.91	26.88	6.57	15.42	30.03	32.66	32.23	3.83	9.56	17.92	17.82	15.41
				TREM2^−/−^	5	Total plaque count/group	1424	2545	4655	4770	4571	1391	2562	4226	4518	4398	972	1951	2766	2835	2835
						Plaque load (%)	9.94	21.24	39.43	41.24	40.67	13.65	21.88	47.34	47.87	47.04	7.12	12.86	24.29	22.81	25.31
3	APP/PS1ΔE9	12 M	Female	TREM2	3	Total plaque count/group	491	1068	2044	2233	2331	414	889	1489	1785	1685	183	548	717	874	965
						Plaque load (%)	4.09	12.58	30.05	30.40	35.16	3.21	11.29	21.13	24.68	23.77	1.87	6.58	9.13	12.23	13.03
				TREM2^T66M^	3	Total plaque count/group	757	1397	2570	2581	2605	735	1349	1975	2073	2060	255	770	1001	1119	1086
						Plaque load (%)	8.02	18.63	39.91	39.44	41.83	7.36	18.89	30.25	31.31	33.08	3.37	12.10	14.19	16.75	15.94

### Immunofluorescence (IF) analysis of mouse brains

Mouse brains were processed as described previously [31, 36, 51]. In brief, 20–25 µm sections were sequentially collected in Phosphate-buffered saline (PBS), placed on charged slides, and stained. For IF staining, Reveal Decloaker (Biocare Medical, #RV1000) was used for antigen retrieval [31] at 95 °C for 30 min. After this, the sections were washed with PBS and subjected to permeabilization with 0.25% Triton X-100 for 20 min before blocking for 2 h in 5% NHS and 3% BSA prepared in 1xPBST (Triton X100-0.2%). Mouse on Mouse Blocking Reagent (Vector laboratories, #MKB-2213) was used for primary antibodies generated in mouse or rat at a dilution of 1 drop/1000 µl. Primary antibodies were diluted in 3%NHS and 1.5% BSA prepared in 1xPBST (Tween 20-0.1%), added on sections and incubated at 4 °C overnight. For TREM2 staining, sections were incubated in the primary antibody at 4 °C for 48 h [31, 51]. After washing steps, appropriate secondary antibodies diluted in 3% NHS and 1.5% BSA prepared in 1xPBST (Tween 20–0.1%) were added on the sections and incubated for 1 h at RT. The sections were then washed and mounted with VECTASHIELD® Hardset™ antifade mounting medium (Vector laboratories, #H-1400) or VECTASHIELD® antifade mounting medium with DAPI (Vector laboratories, #H-1200). Primary and secondary antibodies are summarized in Additional file 1: Table S1.

### Brain protein extraction

Snap-frozen brain hemispheres were extracted as previously described [22, 70]. Briefly, hemispheres were homogenized in PBS, 1 mM EDTA, 1 mM EGTA, 3 μl/ml protease inhibitor mix (Sigma). Homogenates were extracted in RIPA buffer (25 mM Tris–HCl, pH 7.5, 150 mM NaCl, 1% NP40, 0.5% NaDOC, 0.1% SDS), centrifuged at 100,000 g for 30 min and the pellet containing insoluble Aβ was solubilized in 2% SDS, 25 mM Tris–HCl, pH 7.5. The final protein concentration was determined using PierceTM BCA Protein Assay kit (Thermo Fisher) according to the user’s manual.

### Immunoblotting

The brain extracts were separated on 4–12% NuPAGE gels and transferred to 0.45 µm nitrocellulose (NC) membranes as described previously [36]. For immunodetection of proteins, membranes were blocked for 1 h in 5% nonfat dry milk in TBST (Tween 20–0.1%)﻿, then incubated with the primary antibodies in TBST (Tween 20–0.1%) overnight at 4 °C, followed by three washing steps for 10 min with TBST (Tween 20–0.1%)﻿, and addition of appropriate secondary antibodies in TBST (Tween 20–0.1%)﻿. After an incubation period of 60 min at RT, membranes were washed three times for 10 min with TBST (Tween 20–0.1%)﻿, and once for 5 min with TBS. For signal detection, the enhanced chemiluminescence ECL imager (Bio-Rad laboratories, Inc.) or Odyssey CLx™ (LI-COR, Biosciences) were used. The quantification was done by using Image Studio-Lite (Ver. 5.2). Primary and secondary antibodies are summarized in Additional file 1: Table S1.

### Patient material

Tissue samples of patient autopsy cases were provided by the Neurobiobank Munich, Ludwig-Maximilians-University (LMU) Munich. Detailed clinical characteristics were ascertained from an integrated autopsy database. Written informed consent for autopsy and analysis of tissue sample data was obtained for all patients, either from the patients themselves or their kin and the samples were collected according to the guidelines of the local ethics committee following all ethical regulations. Information regarding cases, clinical diagnosis, age at death, post-mortem delay, fixation time, AD Braak & Braak stage, Thal phase, TREM2 coding variant is given in Table 2. The genotyping and identification of TREM2 variant carriers were done as described before [51]. Sample sizes were based on availability of patient material. For all analyses, temporal neocortex was used which included cortex of medial temporal gyrus at the level of anterior hippocampus.

**Table 2:** Demographic and clinical characteristics of the TREM2 coding variants and control (CV) groups. n.i.- no information, ROI- Region of interest. Case#3, with highest fixation time, was omitted for the plaque count and size quantification as it was difficult to define boundaries of extracellular plaques stained with 1E4E11 (pSer8-Aβ) antibody

Case # number	Clinical diagnosis	Age at death (yr)	Sex	Post-mortem Delay (h)	Fixation time (days)	CERAD	Braak & Braak AD stage	Thal phase	ApoE status	TREM2 variant	Total number of Aβ plaques counted/analyzed ROIs	Total number of pSer8-Aβ plaques counted/analyzed ROIs	Total nmAβ intracellular deposits counted/analyzed ROIs	Total pSer8-Aβ intracellular deposits counted/analyzed ROIs
#1	Dementia (rapidly progressive; without further specification)	75	M	24	n.i	C	6	5	E3/E4	R62H (G > A)	13,053	2286	2871	1663
#2	Dementia of the Alzheimer type	78	F	21	15	C	6	5	E3/E4	R62H (G > A)	14,125	2270	2616	1496
#3	Dementia (without further specification)	81	F	11–35	613	C	5	5	E3/E4	R62H (G > A)	17,147	-	2764	2740
#4	Frontotemporal dementia (FTD)	77	M	5	25	C	5	5	E3/E4	R62H (G > A)	15,782	4894	4415	3825
#5	Dementia (without further specification)	86	M	16–40	156	C	5	4	E3/E4	R62H (G > A)	14,296	1984	3731	3675
#6	Frontotemporal dementia (FTD)	77	M	26	297	C	5	4	E3/E4	R62C (C > T)	14,432	1857	3832	2190
#7	Dementia of the Alzheimer type	81	F	60	39	C	6	n.i	E3/E4	None	9384	2057	3304	1052
#13	Dementia, probably of the Alzheimer type	82	M	15	104	C	6	5	E3/E4	None	9816	2759	1850	1841
#14	Dementia (without further specification)	85	M	27	27	C	6	5	E3/E4	None	9892	2386	2157	1021
#15	Dementia (without further specification)	85	M	49	136	C	6	5	E3/E4	None	16,160	4104	2404	2834
#16	Dementia (without further specification)	88	F	9	76	C	4	5	E3/E4	None	14,698	3894	2891	1224
#17	Dementia of the Alzheimer type	75	M	3–13	141	C	5	5	E3/E4	None	8681	2256	2295	837

### Immunohistochemistry (IHC) on human post-mortem brain tissue

IHC was done as described before [51]. In brief, 5 μm temporal neocortex sections were mounted on slides, deparaffinized and rehydrated in a series of xylene and graded ethanol. The sections were subjected to citric acid antigen retrieval (1 M sodium citrate in PBS, pH 6.0) and boiled in a microwave for 20 min. After cooling, endogenous peroxidase activity was quenched using 30% hydrogen peroxide for 20 min. Sections were blocked and incubated with primary antibodies (Additional file 1: Table S1) overnight at 4 °C. Primary antibodies were detected with biotinylated anti-mouse and anti-rat IgG secondary antibodies and visualized with avidin–biotin-complex (ABC-Kit, Vector laboratories) followed by development with diaminobenzidine-HCl (DAB, Vector laboratories) for 5 min. Lastly, sections were counterstained with haematoxylin. Stainings were performed in serially cut sections to compare the same region of interest through all immunostainings. Brightfield images were taken with Axio Scan.Z1 (Carl Zeiss MicroImaging GmbH, Germany).

### Confocal imaging

All IF images were acquired using VisiScope CSU-W1 spinning disk confocal microscope and VisiView Software (Visitron Systems GmbH, Germany). Laser and detector settings were maintained constant for the acquisition of each immunostaining. All stainings were repeated at least three times to ensure reproducibility in the staining protocol. Images were acquired at 10×, 20×, 40×W or 63×W (W-water immersion) objective at 2048 × 2048 pixels, with z-step size of 1 μm (for 10×, 20×, 40×W, 63×W images) or 4-5 μm (for 10× montage image).

### Data analysis of Aβ deposits in mouse and human brain

Initial optimization of dilutions and incubation times for the different antibodies was carried out for detection of the different Aβ species as reported previously [31, 40, 41]. Sections or areas with folds or poor staining quality were excluded from quantifications. To represent and quantify plaque densities (number and size distribution) from three sections/mouse brains regions, the somatosensory cortex (SSC), the retrosplenial cortex (RSC), and the dentate gyrus (DG) (Additional file 1: Figure S1a), acquired images were imported into Fiji software and data channels were separated (image/color/split channels). All layers from a single image stack were projected on a single slice (stack/Z-projection) to test the feasibility of quantifying the area and number of plaques (Additional file 1: Figure S1b). The plaques were then segmented and quantified in Fiji using automatic thresholding methods. Due to the thickness of brain sections and the limited penetration of antibody to plaques underneath the sectioned face, lightly embedded stained plaques may or may not be detected depending on image adjustment values (Additional file 1: Figure S1c). Therefore, the plaques and their quantified combined area in this study constitute the “lower boundary” of the plaque number volume density. Furthermore, as demonstrated with digitalized plaques (SSC region stained with nmAβ as an example (Additional file 1: Figure S2a-b), the number of plaques assessed using an image analysis method in a given brain region varied depending upon the cut-off value of pixel size and the thresholding of plaques. Initial analysis of optical images revealed that plaque-like images that were digitalized often displayed plaque-like artifacts. Therefore, plaques > 10μm^2^ were considered as reliable “digital plaques”. Adjustment of thresholding of certain digitalized plaques appeared to vary in number valuables due to irregularity in plaque shapes or depending upon value of image distribution. It was found that the staining intensity cut-off had a significant impact on the number of plaques. Interestingly, TREM2 knockout mouse brain, showed large size plaques as well as an increased total number of plaques > 10 µm^2^ even at lower thresholding, confirming the validity of the analysis.

For the quantification of plaque ratio in the IF experiments, we considered 300 plaques/region/group (each for SSC and RSC) and 150 plaques/region/group (for DG) (so, SSC = 60 plaques/mouse, RSC = 60 plaques/mouse, DG = 30 plaques/mouse) so making total of 750 plaques/group containing 5 animals or 450 plaques/group containing 3 animals (from at least 3 sections/animal). For this analysis, randomly selected plaques that were positive with all three antibodies detecting Aβ species/staining in 1000 × 1000 µm ROI were analyzed by manually drawing boundary around each plaque by using freehand draw tool in Fiji and determining the IntDen [8] of total Aβ-immunoreactive area for each plaque stained with various antibodies specific to Aβ species within each section for all channels (Additional file 1: Figure S3).

For quantification of microglia surrounding plaques, microglia surrounding 30 cortical plaques of similar plaque area were manually counted and represented as a mean of 30 plaque associated microglia/animal. The total number of neurons was manually counted in a 1000 × 1000 µm area of SSC, in 2 independently stained sections and represented as a ratio of pSer8-Aβ positive neurons/total neurons.

For representation and quantification of plaque densities of 4G8 and pSer8-Aβ stained plaques in the human brain’s sections, 10 cortical 2 × 2 mm regions of interest (ROIs) were randomly selected per case and manually quantified. ROIs were selected to allow analysis of the same region for all four consecutive brain sections stained with different antibodies without interference by cuts, folds, or other irregularities (Additional file 2: Source data 1). The total number of plaques along with area measurements were considered for the analysis. The border around plaques was manually drawn by using “spline contour” tool in the ZEN 3.2 software, for the area stained with different antibodies. With 4G8 antibody-stained sections, an area cutoff of 10 µm^2^ was determined, while with pSer8-Aβ antibody-stained sections, an area cutoff of 30 µm^2^ was determined, below which were considered as either artifacts or intracellular deposits (quantified separately). Case#3, with highest fixation time, was omitted for the plaque count and size quantification as it was difficult to define boundaries of extracellular plaques stained with 1E4E11 (pSer8-Aβ) antibody. Aβ plaque load was calculated by summing the areas of all counted plaques divided by the total area of all ROIs. The intracellular deposits of nmAβ and phosphorylated Aβ were manually counted from 10 cortical consecutive 2 × 2 mm randomly selected ROIs per case.

### Statistical analysis

Statistical analyses were performed using GraphPad prism software. The plaque area distribution showed positively skewed distribution [58] and varied from the normal distribution confirmed by D’Agostino & Pearson and the Shapiro–Wilk normality test. Hence, for this analysis, we considered non-parametric, Mann Whitney test (compares the distributions of ranks in two groups) and Kolmogorov–Smirnov test (compares the cumulative distributions) for the frequency distribution of all values. Besides this, since the data were not formally tested, we assumed it followed Gaussian distribution and that the variance between groups was comparable [51]; thus, unless otherwise stated, two-sided, unpaired t-test with Welch's correction was used to determine the statistical difference between groups in analyses requiring only single comparisons. The degree of significance between groups is represented as *p < 0.05, **p < 0.01, ***p < 0.001, ****p < 0.0001, and ^ns^*p* > 0.05.

#### Randomization and blinding

The immunohistochemical analysis of mouse and human brains was initially performed blinded with coded slides. However, complete randomization was not possible in the staining with a microglial marker or when stained with anti-TREM2 antibody [31, 51], depicting microglial clustering. No randomization procedure was performed for selecting patient material as case inclusion was largely based on availability. Following the completion of the analysis, the groups were unblinded to perform statistics.

#### Data collection

Confocal images were acquired by using VisiScope CSU-W1 spinning disk confocal microscope and VisiView Software (Visitron Systems GmbH, Germany). Human brain immunostaining data were acquired by Axio Scan.Z1 at Plan-Apochromat 20x/0.8M27 objective imaged by Hitachi HV-F202SCL with ZEN 3.2 software (Carl Zeiss MicroImaging GmbH, Germany). FIJI (ImageJ) or ZEN 3.2 software was used for all immunohistochemical analyses. Microsoft Excel was used to organize and to calculate the averages of each repeated experiment. GraphPad (Prism v7.0) software was used to build graphs and perform statistical analyses presented throughout the manuscript.

#### Validation

Phosphorylation-state specific antibodies were generated and validated as described previously [38, 41]. Antibody 1E4E11 is specific for pSer8-Aβ and does not cross-react with other post-translationally modified variants of Aβ, including N-terminally truncated (Aβ 3–42), nitrated (3NTyr10-Aβ), pyroglutamated (N3pE-Aβ), or Aβ phosphorylated at Ser 26 [41]. Rabbit polyclonal antibody 2964 was raised against aggregated Aβ [73]. TREM2 antibody was verified for immunostainings [28, 51]. 7H3D6 antibody specifically detects Aβ species with a non-modified N-terminus and does not recognize phosphorylated Ser8-Aβ, but also does not recognize other Aβ variants with N-terminal modifications, including pyroglutamated, nitrated, and N-terminally truncated Aβ species [41]. It is important to note that both antibodies 1E4E11, detecting pSer8-Aβ and 7H3D6 used for detection of N-terminally non-modified Aβ, do not cross-react with full-length APP or APP C-terminal fragments [40, 41]. All the other antibodies used in the study were verified for immunostaining and immunoblotting in mouse and human samples according to the company websites (Additional file 1: Table S1).

## Results

### Selective accumulation of Ser8-phosphorylated Aβ species upon loss of TREM2 function in brains of transgenic mice

Phosphorylated Aβ variants were previously detected in brains of transgenic mouse models and human AD cases, and shown to exert increased toxicity in Drosophila models and human neurons derived from embryonic stem or induced pluripotent stem cells [11, 13, 38, 40, 41, 54]. To assess the role of TREM2 in the deposition of modified Aβ species in-vivo, 5xFAD transgenic mice were crossed with TREM2^+/+^ or TREM2^−/−^ mice as described previously [31, 51] (Table 1), and the deposition of different Aβ species was analyzed using several antibodies selectively detecting modified and non-modified variants of Aβ. Monoclonal antibody 1E4E11 specifically detects pSer8-Aβ species, while monoclonal antibody 7H3D6 selectively recognizes Aβ with Ser8 in non-phosphorylated state. Antibody 7H3D6 also does not recognize other Aβ variants with N-terminal modifications, including pyroglutaminated, nitrated, and N-terminally truncated Aβ species [41].

**Fig. 1:**
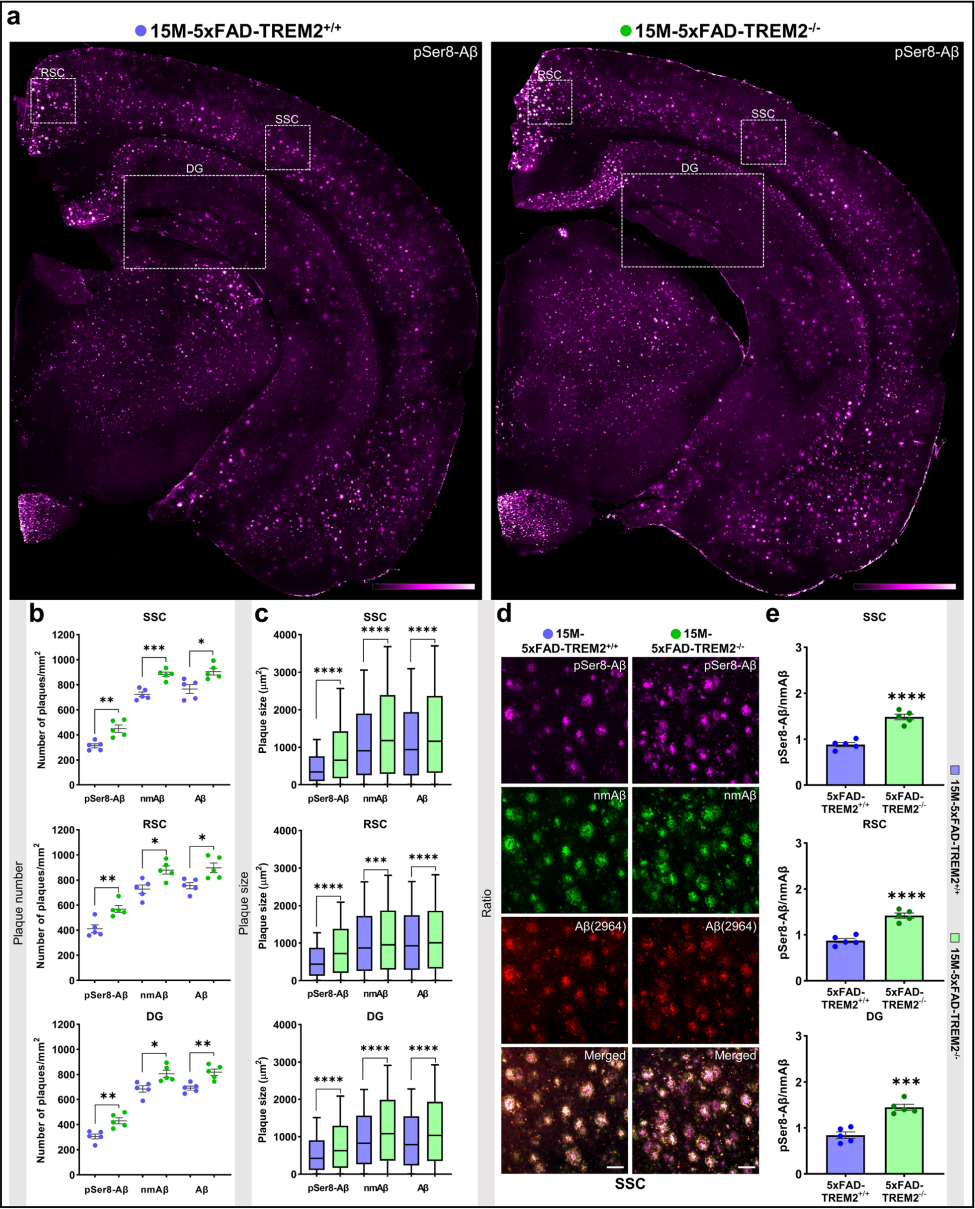
TREM2 deletion leads to increased deposition of pSer8-Aβ in 15 M old 5xFAD transgenic mouse brains. **a** Representative pSer8-Aβ stained male 15 M old-5xFAD-TREM2^+/+^ and TREM2^−/−^ mouse brain sections (color scale bar = 35 mm, represents min/max pixel intensities, 10x). **b** Dot plots representing number of plaques/mm^2^, **c** Box and whiskers plots showing plaque size (µm^2^) stained with pSer8-Aβ, nmAβ and Aβ (2964) antibodies in the SSC, RSC, and DG of male 15 M-5xFAD-TREM2^−/−^ and  TREM2^+/+^ mice. **d** Representative images showing deposition of pSer8-Aβ, nmAβ, and Aβ (2964) in SSC of male 15 M-5xFAD-TREM2^−/−^ and  TREM2^+/+^ mice (scale bar = 50 µm, 40xW). **e** Ratios of pSer8/nmAβ in SSC (*t*(7.41) = 7.856,****p < 0.0001), RSC (*t*(7.696) = 7.611,****p < 0.0001), and DG (*t*(7.945) = 6.345,***p = 0.0002) of male 15 M-5xFAD-TREM2^−/−^ compared with TREM2^+/+^ mice. Each dot represents average value of the number of plaques or the ratio of pSer8-Aβ/nmAβ per animal. The box and whiskers plots represent min/max values of distribution of plaque size with the median (shown by the line dividing the box) and the dot plots represent mean ± SEM (n = 5 animals, color- blue (5xFAD-TREM2^+/+^) and green (5xFAD-TREM2^−/−^), unpaired t-test with Welch's correction for analysis of the number and ratio while Mann–Whitney test for plaque size, ^ns^*p* > 0.05, *p < 0.05, **p < 0.01, ***p < 0.001 or ****p < 0.0001)

**Fig. 2:**
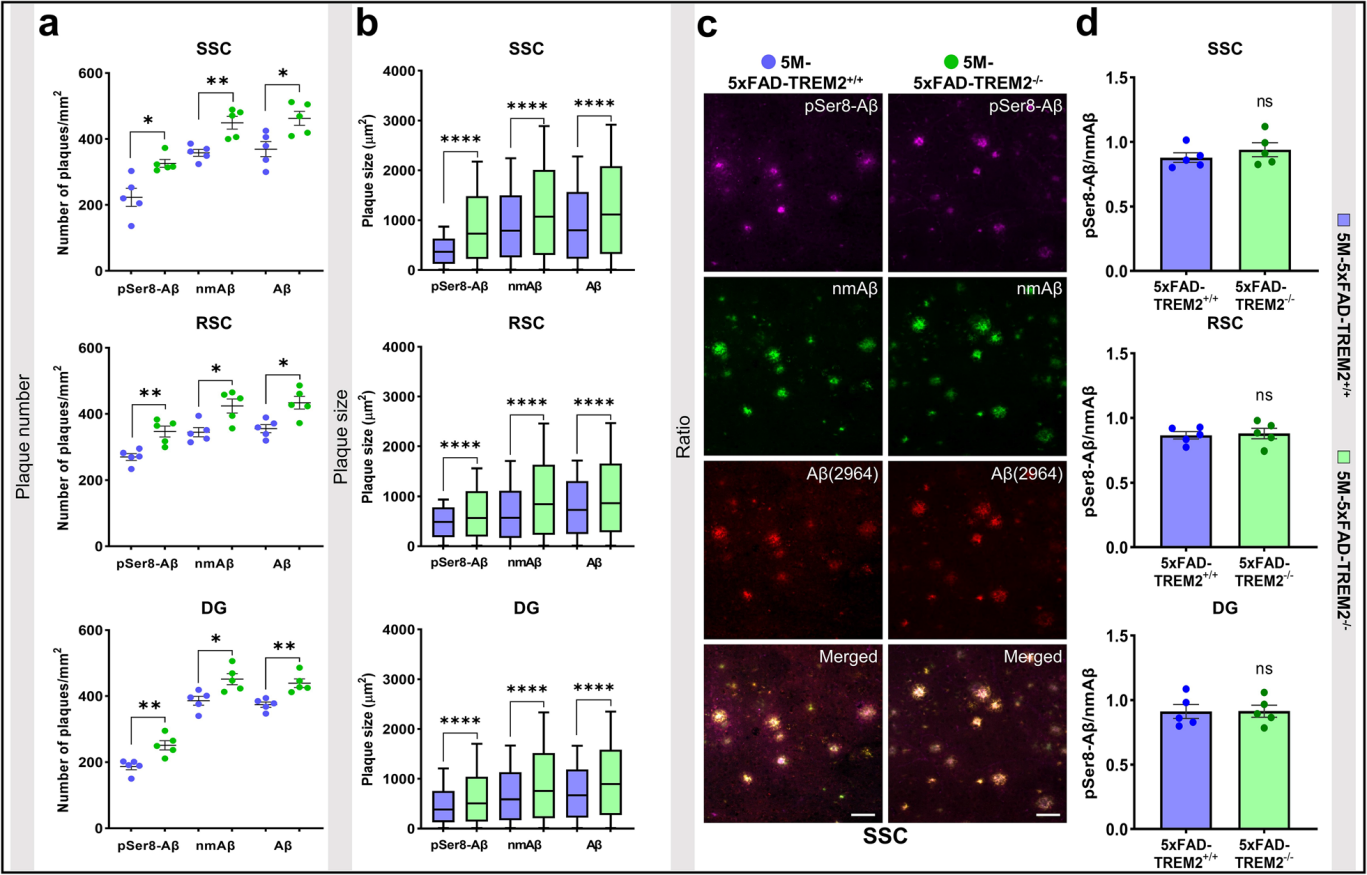
TREM2 deletion leads to increased deposition of pSer8-Aβ in 5 M old 5xFAD transgenic mouse brains. **a** Dot plots representing number of plaques/mm^2^, **b** Box and whiskers plots showing  plaque size (µm^2^) stained with pSer8-Aβ, nmAβ and Aβ (2964) antibodies in the SSC, RSC, and DG of male 5 M old-5xFAD-TREM2^−/−^ and  TREM2^+/+^ mice. **c** Representative images showing deposition  of pSer8-Aβ, nmAβ, and Aβ (2964) in SSC of male 5 M-5x-FAD-TREM2^−/−^ and  TREM2^+/+^ mice (scale bar = 50 µm, 40xW). **d** Ratio of pSer8/nmAβ in SSC, RSC and DG in male 5 M-5xFAD-TREM2^−/−^ compared to TREM2^+/+^ mice. Each dot represents average value of the number of plaques or the ratio of pSer8-Aβ/nmAβ per animal. The box and whiskers plots represent min/max values of distribution of plaque size with the median (shown by the line dividing the box) and the dot plots represent mean ± SEM (n = 5 animals, color- blue (5xFAD-TREM2^+/+^) and green (5xFAD-TREM2^−/−^), unpaired t-test with Welch's correction for analysis of the number and ratio while Mann–Whitney test for plaque size, ^ns^*p* > 0.05, *p < 0.05, **p < 0.01 or ****p < 0.0001)

Triple staining with mouse monoclonal 1E4E11, rat monoclonal 7H3D6, and rabbit polyclonal 2964 antibodies revealed that TREM2^−/−^ mice at 15 months (15 M) of age had significantly more plaques detected by all three antibodies. Elevated plaque deposition, as measured by the plaque number (Fig. 1a–b, Additional file 1: Figure S4) and plaque load (Table 1), was detected in the three different brain regions analyzed, the somatosensory cortex (SSC), the retrosplenial cortex (RSC), and the dentate gyrus (DG). Consistent with a preferential deposition in the core of plaques, pSer8-Aβ positive deposits are of smaller size than those containing nmAβ that is also deposited in the corona of plaques (Fig. 1c, Additional file 2: Source data 2a-c). These findings are consistent with the function of TREM2 to restrict plaque size or growth [75, 81], and also demonstrate the importance of TREM2 to limit accumulation of pSer8-Aβ in the core of plaques. A selective increase in pSer8-Aβ was detected by analyzing the mean fluorescence signal intensities within Aβ deposits (Fig. 1d–e). Furthermore, increased number and size of pSer8-Aβ and nmAβ positive plaques in 5xFAD TREM2^−/−^ compared to TREM2^+/+^ mouse brains was already observed in 5 M old mice (Fig. 2a–b, Additional file 2: Source data 2d-f). Analysis of plaque size distribution at both ages revealed overall increased numbers of deposits of various sizes in all three analyzed brain regions of TREM2^−/−^ mice (Additional file 2: Source data 2). In particular, the number of deposits > 1200 µm^2^ that are also positive for pSer8-Aβ were strongly increased in TREM2^−/−^ mice already at 5 M of age, suggesting that TREM2 decreases the formation and growth of plaques already at early stages of deposition. However, the ratio of the mean fluorescence intensity for pSer8-Aβ and nmAβ was similar in TREM2^−/−^ and TREM2^+/+^ at this young age (Fig. 2c–d), indicating that TREM2 deficiency promotes co-deposition of different Aβ species. Very similar findings were obtained with an independent double-transgenic APP/PS1L166P mouse model (Table 1, Additional file 1: Figure S5, Additional file 2: Source data 3).

**Fig. 3:**
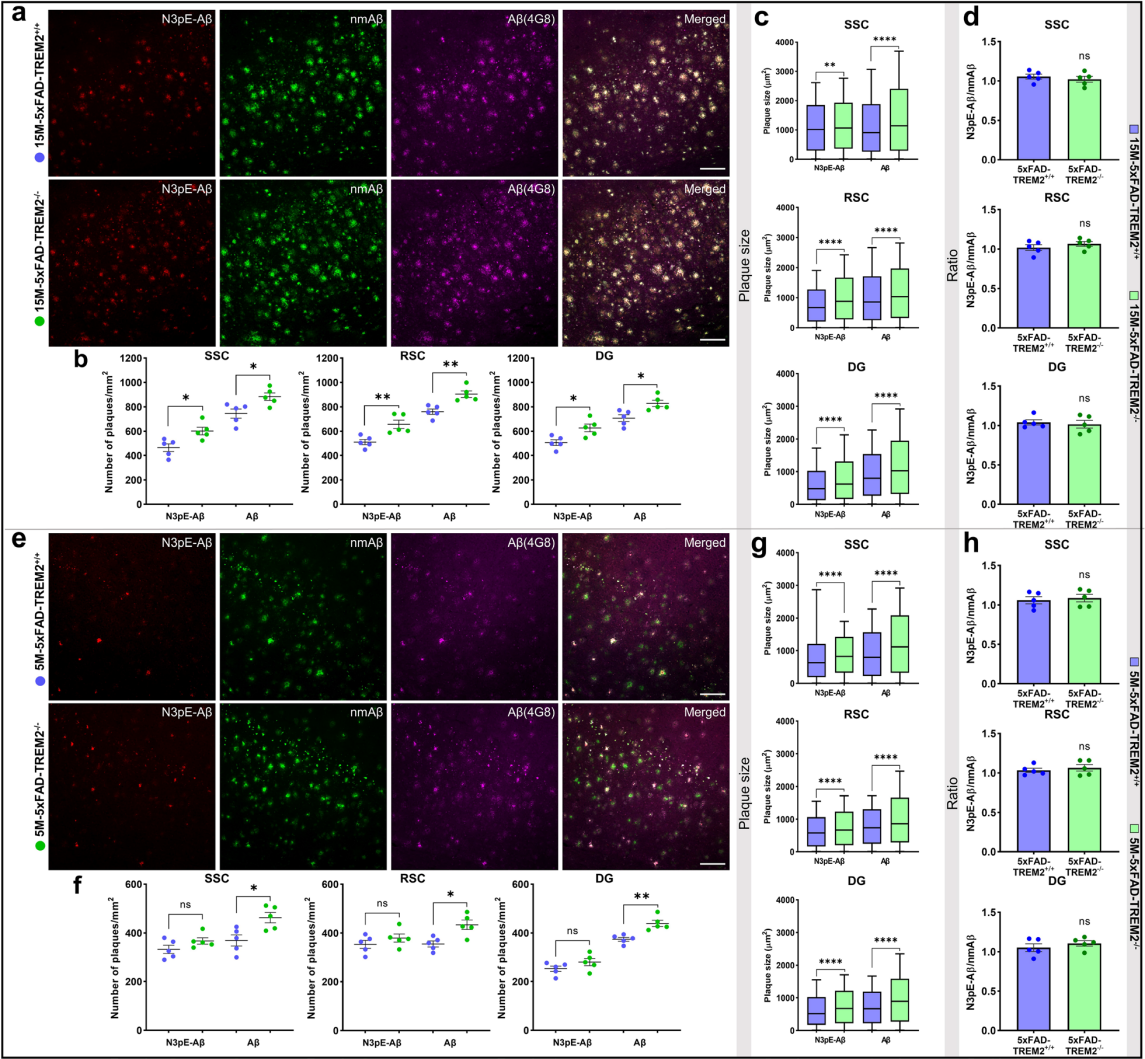
TREM2 deletion leads to increased N3pE-Aβ deposits in 5xFAD transgenic mouse brains. **a** Representative images showing deposition of N3pE-Aβ, nmAβ, and Aβ (4G8) in SSC of male 15 M old-5xFAD-TREM2^−/−^ and  TREM2^+/+^ mice (scale bar = 200 µm, 20x). **b** Dot plots representing  number of plaques/mm^2^, **c** Box and whiskers plots showing plaque size (µm^2^) stained with N3pE-Aβ and Aβ(4G8) antibodies in the SSC, RSC, and DG of the male 15 M-5xFAD-TREM2^−/−^ and  TREM2^+/+^ mice. **d** Ratio of N3pE-Aβ/nmAβ in the SSC, RSC, and DG of male 15 M-5xFAD-TREM2^−/−^ compared with TREM2^+/+^ mice. **e** Representative images showing deposition of N3pE-Aβ, nmAβ, and Aβ (4G8) in SSC of male 5 M-5xFAD-TREM2^−/−^ and  TREM2^+/+^ mice (scale bar = 200 µm, 20x). **f** Dot plots representing number of plaques/mm^2^, **g** Box and whiskers plots showing plaque size (µm^2^) stained with N3pE-Aβ and Aβ (4G8) antibodies in the SSC, RSC, and DG of male 5 M-5xFAD-TREM2^−/−^ and  TREM2^+/+^ mice. **h** Ratio of N3pE-Aβ/nmAβ in the SSC, RSC, and DG of male 5 M-5xFAD-TREM2^−/−^ compared with TREM2^+/+^ mice. Each dot represents average value of the number of plaques or the ratio of N3pE-Aβ/nmAβ per animal. The box and whiskers plots represent min/max values of distribution of plaque size with the median (shown by the line dividing the box) and the dot plots represent mean ± SEM (n = 5 animals, color- blue (5xFAD-TREM2^+/+^) and green (5xFAD-TREM2^+/+^), unpaired t-test with Welch's correction for analysis of the number and ratio while Mann–Whitney test for plaque size, ^ns^*p* > 0.05, *p < 0.05, **p < 0.01 or ****p < 0.0001)

Pyroglutamate-modified Aβ (N3pE-Aβ) also accumulates during the pathogenesis of AD in the cortex and hippocampus [52, 55]. Immunohistochemical analyses showed that TREM2^−/−^ mice had significantly more N3pE-Aβ positive plaques at 15 M of age in the SSC, RSC, and the DG as compared to TREM2^+/+^ mice. Consistent with data shown in Fig. 1, TREM2^−/−^ mice showed higher number of plaques, (Fig. 3a-b), increased size of plaques (Fig. 3c, Additional file 2: Source data 2a-c) and elevated plaque load (Table 1) in all three regions as revealed by co-staining with antibodies detecting N-terminally non-modified Aβ and total Aβ species at 15 M of age. In contrast to pSer8-Aβ, N3pE-Aβ was not selectively increased  in plaques as compared to nmAβ at 15 M of age, as evidenced by similar ratios of N3pE-Aβ/nmAβ in all analyzed brain regions (Fig. 3d). At 5 M of age, the total number of N3pE-Aβ deposits were only slightly, but not significantly increased in 5xFAD TREM2^−/−^ mice as compared to TREM2^+/+^ 5xFAD mice (Fig. 3e–f). However, as observed with 15 M old mice, there was a significant increase in the size of N3pE-Aβ positive plaques especially for larger deposits > 1500 µm^2^ already at 5 M in 5xFAD TREM2^−/−^ mice (Fig. 3g, Additional file 2: Source data 2d–f). Analysis of plaque size distribution revealed that there was an increase in the number of N3pE-Aβ deposits at both age groups, again particularly pronounced for deposits > 1500 µm^2^. An increase of pSer8-Aβ and N3pE-Aβ deposits < 600 µm^2^ in the three analyzed brain regions of TREM2^−/−^ mice as compared to TREM2^+/+^ mice was only observed at 15 M of age (Additional file 2: Source data 2). The N3pE-Aβ/nmAβ intensity ratio in the analyzed brain regions was not different between TREM2^−/−^ and TREM2^+/+^ mice at both ages (Fig. 3d, h). Very similar findings were obtained with the independent double-transgenic APP/PS1L166P mouse model (Additional file 1: Figure S6, Additional file 2: Source data 3). These findings indicate that N3pE-Aβ species showed increased deposition upon deletion of TREM2. However, N3pE-Aβ, in contrast to pSer8-Aβ, did not selectively accumulate upon deletion of TREM2 in relation to nmAβ.

### TREM2^T66M^ mutation leads to increased deposition of pSer8-Aβ in transgenic mouse brain

**Fig. 4:**
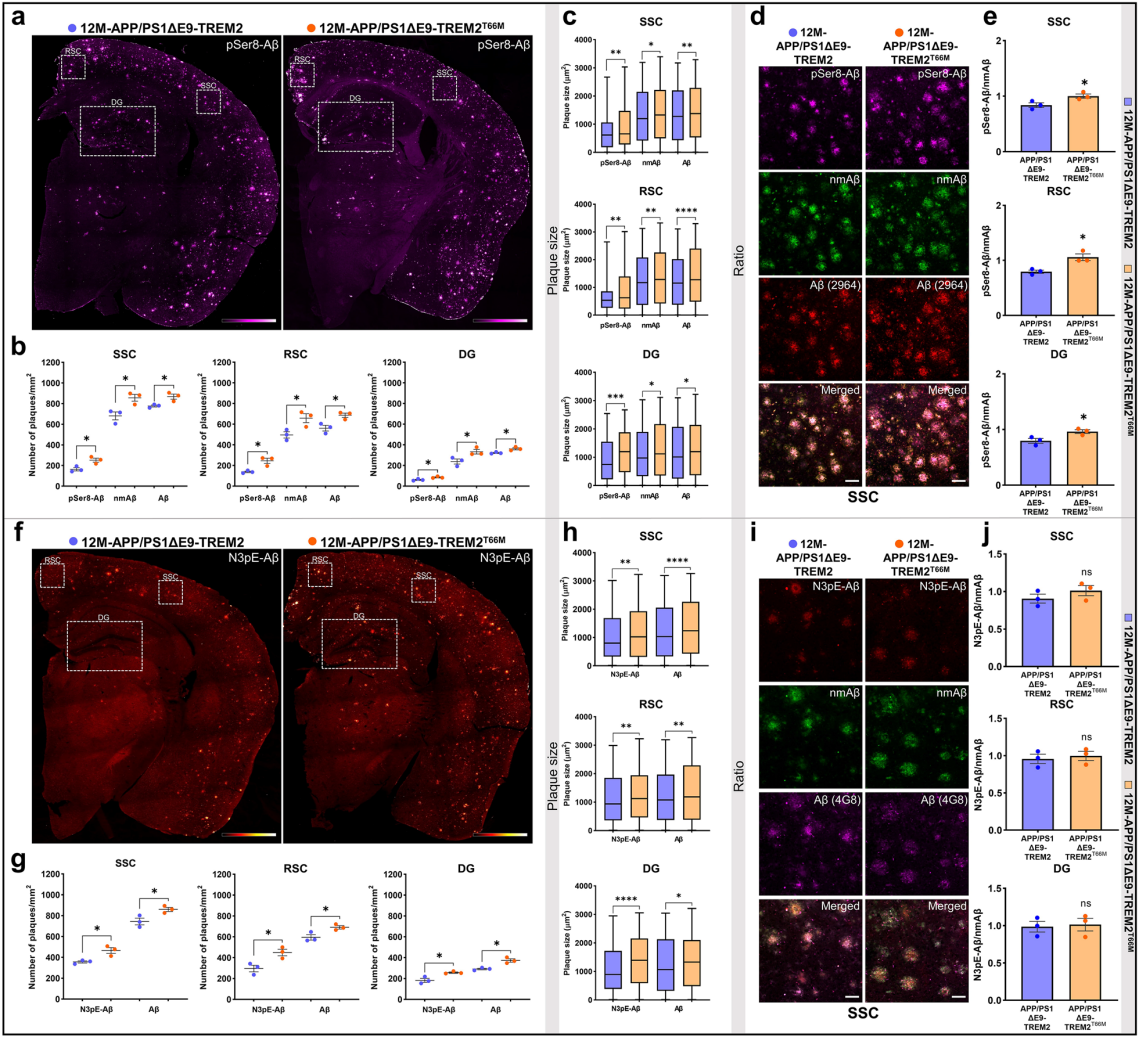
TREM2^T66M^ causes increased deposition of pSer8-Aβ and N3pE-Aβ in APP/PS1 transgenic mouse brains. **a** Representative pSer8-Aβ stained female 12 M old-APP/PS1ΔE9-TREM2 and APP/PS1ΔE9-TREM2^T66M^ mouse brain sections (color scale bar = 35 mm, represents min/max pixel intensities, 10x). **b** Dot plots representing number of plaques/mm^2^, **c** Box and whiskers plots showing sizes of plaques (µm^2^) stained with N3pE-Aβ, nmAβ, and Aβ (2964) antibodies in the SSC, RSC, and DG of female 12 M APP/PS1ΔE9-TREM2^T66M^ and APP/PS1ΔE9-TREM2 mice. **d** Representative images showing deposition of pSer8-Aβ, nmAβ, and Aβ (2964) in SSC of female 12 M-APP/PS1ΔE9-TREM2^T66M^  and APP/PS1ΔE9-TREM2 mice (scale bar = 50 µm, 40xW). **e** Ratio of pSer8/nmAβ in SSC (*t*(3.953) = 2.863, *p = 0.0464), RSC (*t*(2.915) = 3.94,*p = 0.0307), and DG (*t*(3.743) = 3.018,*p = 0.0427) of female 12 M-APP/PS1ΔE9-TREM2^T66M^ compared with APP/PS1ΔE9-TREM2 mice. **f** Representative N3pE-Aβ stained female 12 M-APP/PS1ΔE9-TREM2 and APP/PS1ΔE9-TREM2^T66M^ mouse brain sections (color scale bar = 35 mm, represents min/max pixel intensities, 10x). **g** Dot plots representing number of plaques/mm^2^, **h** Box and whiskers plots representing size of plaques (µm^2^) stained with N3pE-Aβ and Aβ (4G8) antibodies in the SSC, RSC, and DG of the female 12 M-APP/PS1ΔE9-TREM2^T66M^ and APP/PS1ΔE9-TREM2 mice. **i** Representative images showing deposition of N3pE-Aβ, nmAβ, and Aβ (4G8) in SSC of female 12 M-APP/PS1ΔE9-TREM2^T66M^  and APP/PS1ΔE9-TREM2 mice (scale bar = 50 µm, 40xW). **j** Ratio of N3pE-Aβ/nmAβ in SSC, RSC, and DG of female 12 M-APP/PS1ΔE9-TREM2^T66M^ compared with APP/PS1ΔE9-TREM2 mice. Each dot represents average value of number of plaques or ratio/animal. The box and whiskers plots represent min/max values of distribution of plaque size with the median (shown by the line dividing the box) and the dot plots represent mean ± SEM (n = 3 animals, color- blue (APP/PS1ΔE9-TREM2) and orange (APP/PS1ΔE9-TREM2^T66M^), unpaired t-test with Welch's correction for analysis of the number and ratio while Mann–Whitney test for plaque size, ^ns^*p* > 0.05, *p < 0.05, **p < 0.01, ***p < 0.001 or ****p < 0.0001)

To assess potential effects of a disease associated TREM2 mutant, we used knock-in (KI) mice that carry the TREM2^T66M^ variant (Table 1). The TREM2^T66M^ variant is associated with Nasu–Hakola disease (NHD) and frontal lobe degeneration [17, 19, 49]. Importantly, the genetic modification at this site in the mouse genome does not result in aberrant splicing and lower mRNA levels as observed previously for knock-in mice expressing the Alzheimer-associated R47H variant [79]. TREM2^T66M^ KI mice were crossed with APP/PS1ΔE9 double transgenic mice. Mice homozygous for endogenous TREM2 or the TREM2^T66M^ KI mutation were analyzed at 12 M of age. Homozygous TREM2^T66M^ KI mice showed an increase in the number and size of plaques (Fig. 4a–c, Additional file 2: Source data 4) as well as increased plaque load (Table 1). TREM2^T66M^ KI mice also showed a selective accumulation of pSer8-Aβ in plaques as compared to nmAβ (Fig. 4d–e). Moreover, there was an increase in the number of larger sized (> 1500 µm^2^) as well as smaller sized (< 600 µm^2^) deposits of N3pE-Aβ along with pSer8-Aβ, nmAβ and total Aβ species in the analyzedbrain regions of TREM2^T66M^ mice (Fig. 4f–h, Additional file 2: Source data 4). Again, no selective accumulation of N3pE-Aβ as compared to that of nmAβ was observed in brains of TREM2^T66M^ expressing mice (Fig. 4i–j). These findings further support a selective increase of pSer8-Aβ in parenchymal plaques in mice with impaired TREM2 function.

### Loss of TREM2 function increases intraneuronal and vascular deposition of Aβ species

**Fig. 5:**
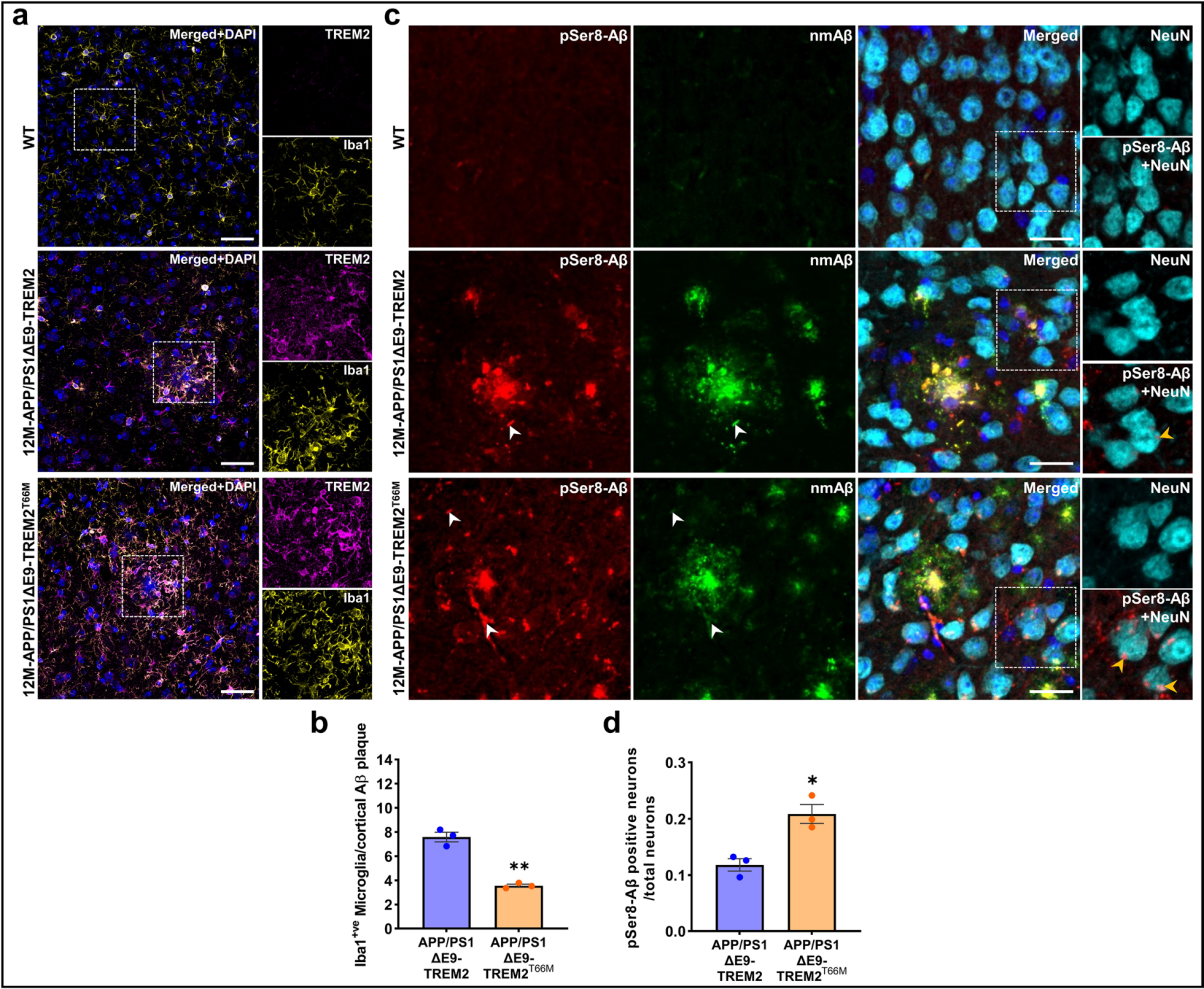
TREM2^T66M^ causes increased Aβ in the APP/PS1 transgenic mouse brains. **a** Representative IF images of TREM2 along with microglial marker, Iba1 in female 12 M old- APP/PS1ΔE9 and APP/PS1ΔE9-TREM2^T66M^ mice. Dotted white boxes indicate the area shown at higher magnification (Scale bar = 50 μm, 20x). **b** Quantification showing decreased plaque associated Iba1^+ve^ microglia surrounding cortical plaques in female 12 M-APP/PS1ΔE9-TREM2^T66M^ as compared to APP/PS1ΔE9-TREM2 mice (*t*(2.392) = 9.566,**p = 0.0058). Each dot represents the mean value of microglia count surrounding 30 cortical plaques/animal. **c** Representative IF images of pSer8-Aβ and nmAβ localized in extracellular plaques,  vessels (white arrowheads) and within NeuN positive neurons (yellow arrowheads) in SSC of female APP/PS1ΔE9-TREM2 and APP/PS1ΔE9-TREM2^T66M^ mice. Dotted white boxes indicate the area shown at higher magnification (Scale bar = 50 μm, 40xW). **d** Quantification of intraneuronal pSer8-Aβ normalized to total number of neurons/ROI showed significantly increased intraneuronal pSer8-Aβ deposits in the SSC of female 12 M-APP/PS1ΔE9-TREM2^T66M^ as compared to APP/PS1ΔE9-TREM2 mice (*t*(3.466) = 4.459, *p < 0.0154). All data represent mean ± SEM (n = 3 animals, color- blue (APP/PS1ΔE9-TREM2) and orange (APP/PS1ΔE9-TREM2^T66M^), unpaired t-test with Welch's correction)

Clustering of Iba1 positive microglia around plaques was apparent in the different APP transgenic mouse models expressing endogenous TREM2 at advanced and earlier stages of Aβ deposition, but was strongly reduced in brains of the respective TREM2^−/−^ (Additional file 1: Figure S7a-b) and TREM2^T66M^ KI mice (Fig. 5a-b). Consistent with previous reports [8, 28, 51], TREM2^−/−^ mice showed much less compact Aβ deposits as compared to TREM2^+/+^ mice. Similar observations were made with the APP/PS1ΔE9-TREM2^T66M^ KI mice as compared to APP/PS1ΔE9-TREM2 mice, indicating that impaired barrier function of microglia caused by dysfunctional TREM2 promotes the deposition of smaller plaques containing pSer8-Aβ. In addition, brains of APP transgenic TREM2^−/−^ and TREM2^T66M^ KI mice showed strongly elevated deposition of pSer8-Aβ within neurons as compared to brains from APP transgenic mice expressing endogenous functional TREM2 (Fig. 5c–d, Additional file 1: Figure S7c-d).

**Fig. 6:**
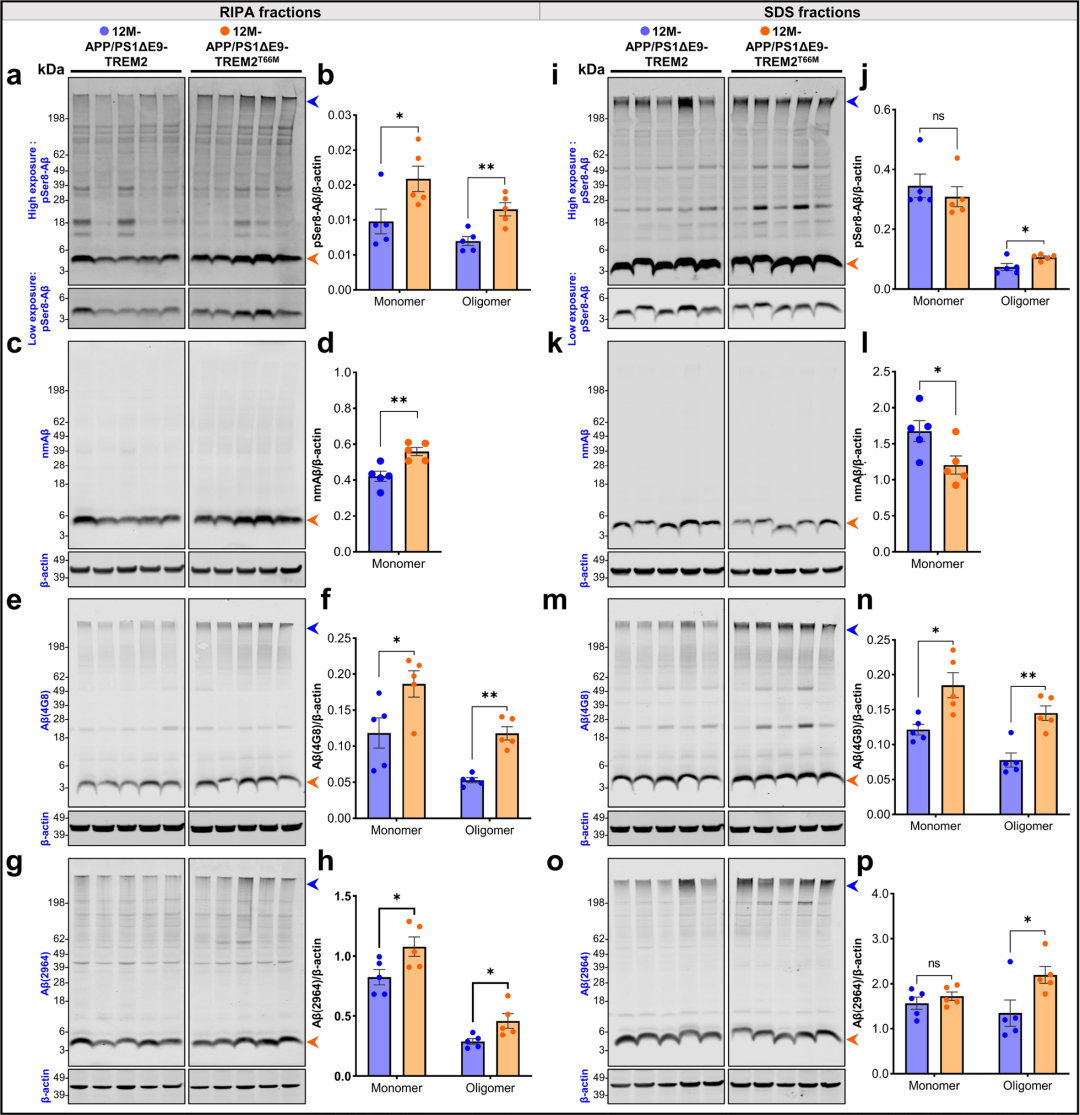
Increased pSer8-Aβ in brain extracts of APP/PS1 ΔE9-TREM2^T66M^ transgenic mouse brains. **a–h** Immunoblots and quantification showing levels of monomeric and oligomeric Aβ variants in RIPA extracts of brains of female 12 M old-APP/PS1ΔE9-TREM2^T66M^ as compared to APP/PS1ΔE9-TREM2 mice; pSer8-Aβ immunostained with antibody 1E4E11, (*monomer: t*(7.993) = 2.391, *p = 0.0438; *oligomer: t*(7.074) = 3.925, **p = 0.0056), nmAβ detected with antibody 7H3D6 (*monomer: t*(7.608) = 3.849, **p = 0.0054), Aβ immunostained with antibody 4G8 (*monomer: t*(7.847) = 2.454, *p = 0.0402; *oligomer: t*(5.134) = 6.604, **p = 0.0011) or antibody 2964 (*monomer: t*(7.607) = 2.46, *p = 0.0408; *oligomer: t*(5.181) = 2.63, *p = 0.045) antibodies. **i–p** Immunoblots and quantification showing levels of monomeric and oligomeric Aβ variants in SDS extracts of brains of female 12 M-APP/PS1ΔE9-TREM2^T66M^ as compared to APP/PS1ΔE9-TREM2 mice; pSer8-Aβ immunostained with antibody 1E4E11, (*monomer: t*(7.81) = 0.708, ^ns^*p* = 0.4995; *oligomer: t*(5.14) = 2.62, *p = 0.0458), nmAβ detected with antibody 7H3D6 (*monomer: t*(7.886) = 2.449, *p = 0.0404), Aβ immunostained with antibody 4G8 (*monomer: t*(5.396) = 3.255, *p = 0.0202; *oligomer: t*(7.999) = 4.642, **p = 0.0017) or antibody 2964 (*monomer: t*(7.153) = 0.939, ^ns^*p* = 0.3784; *oligomer: t*(6.775) = 2.434, *p = 0.0463) antibodies. Each dot represents ratio of Aβ signal to β-actin per animal. All data represent mean ± SEM (n = 5 animals, *monomer: orange arrowheads and oligomer: blue arrowheads*, color- blue (APP/PS1ΔE9-TREM2) and orange (APP/PS1ΔE9-TREM2^T66M^), unpaired t-test with Welch's correction). Original uncropped immunoblots are provided in the Additional file 2: Source data 5

We also performed western immunoblotting analyses with different fractions of brain extracts from APP/PS1ΔE9-TREM2 and APP/PS1ΔE9-TREM2^T66M^ KI mice. Levels of pSer8-Aβ, nmAβ, and total Aβ (immunostained with 4G8 or 2964 antibody) were significantly increased in the RIPA extracts with low detergent concentration that could contain extracellular and membrane-associated monomeric and oligomeric Aβ species (Fig. 6a–h). Consistent with the higher aggregation propensity of pSer8-Aβ, pSer8-Aβ reactivity was also observed in the upper region of the blot that likely represent SDS-stable oligomers. These species were also detected by antibodies 4G8 or 2964 that detect Aβ independently of the modification state. In contrast, non-modified Aβ was almost exclusively detected as monomers. However, levels of monomeric non-modified Aβ were also increased in RIPA extracts of APP/PS1ΔE9-TREM2^T66M^ KI as compared to that of APP/PS1ΔE9-TREM2 WT mouse brains. In the SDS fractions that were obtained subsequently after extraction with RIPA buffer, and could also contain intracellular Aβ, levels of pSer8-Aβ migrating in the monomeric band were similar between TREM2 WT and TREM2^T66M^ brains. Notably, levels of oligomeric pSer8-Aβ were elevated in TREM2^T66M^ brains, indicating higher levels of pSer8-Aβ containing oligomers upon loss of TREM2 function. As observed for the RIPA extracts, nmAβ was also not detected as oligomers in the SDS fraction. Levels of monomeric nmAβ were decreased in the SDS fraction of TREM2^T66M^ mice as compared to that of TREM2 WT mice (Fig. 6i–l). Increased levels of oligomeric Aβ in brains of TREM2^T66M^ mice were confirmed with antibodies 4G8 and 2964 in both, RIPA and SDS extracts (Fig. 6m–p). Together these data indicate that TREM2 could limit the accumulation of oligomeric Aβ assemblies which contain Ser8-Aβ. These oligomers might further aggregate to form Aβ deposits consistent with the increased plaque deposition observed in brains of mice with TREM2 deletion or expression of the dysfunctional TREM2^T66M^ variant. Notably, increased deposition of pSer8-Aβ and nmAβ was not only observed in form of extracellular plaques, but also in brain vessels of APP/PS1L166P-TREM2^-/-^ mice as compared to APP/PS1L166P-TREM2^+/+^ mice (Additional file 1: Figure S7e). This vascular deposition of Aβ resembles cerebral amyloid angiopathy (CAA) observed in different APP transgenic mouse models, and very commonly in human AD brains [11, 37, 65].

### TREM2 variants are associated with quantitative and qualitative differences in the deposition of distinct Aβ species in human brains

**Fig. 7:**
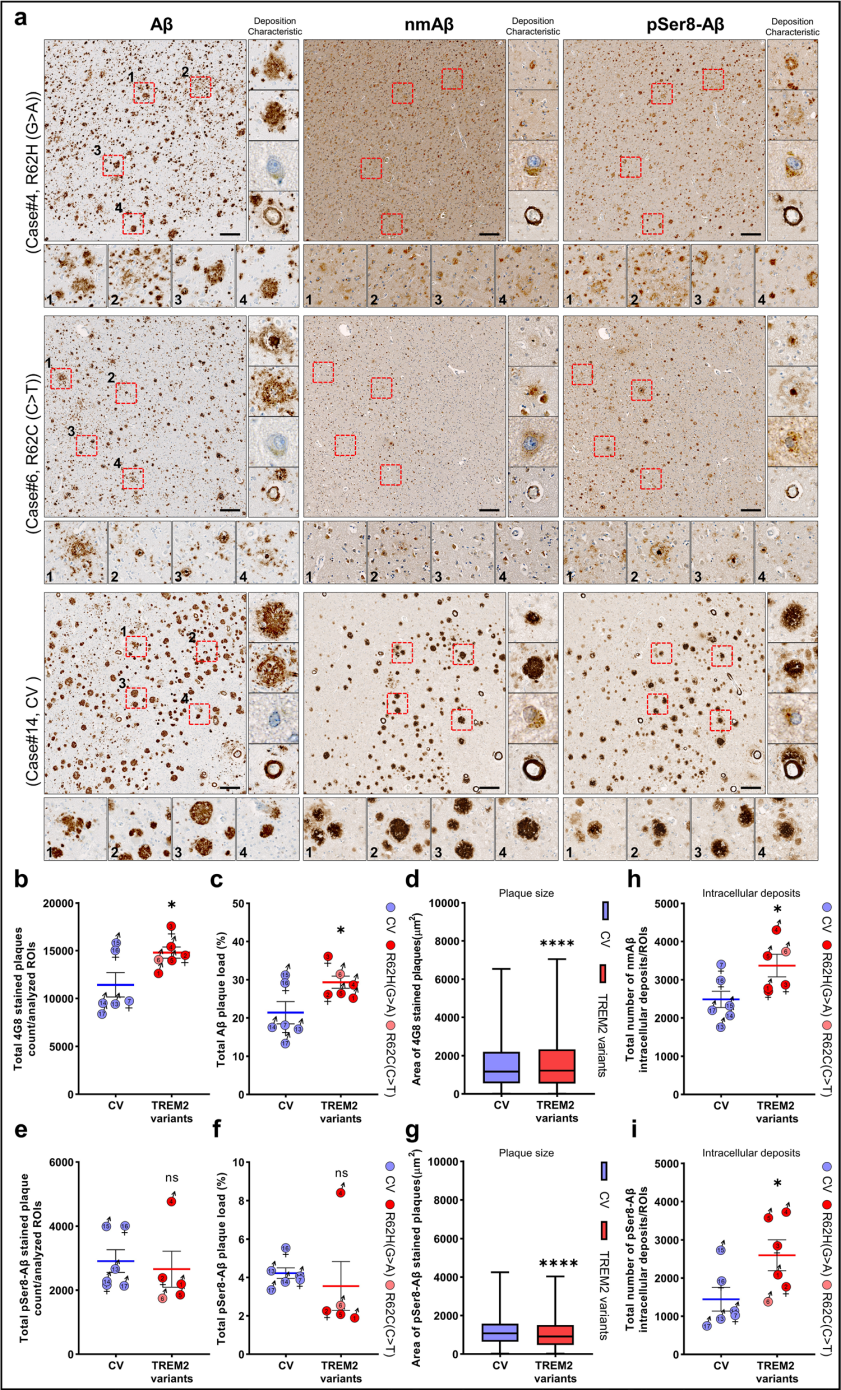
Differential deposition of phosphorylated Aβ species in human brains with the common and rare variants of TREM2. **a** Temporal neocortex of human AD patients with the indicated TREM2 variants (see Table 2) stained to detect total Aβ (with 4G8), nmAβ, and pSer8-Aβ on consecutive sections. Immunostaining clearly indicated decreased deposition of N-terminally non-modified Aβ species in the TREM2 variants. pSer8-Aβ stained the plaques along with vascular as well as intracellular deposits. (Scale bar = 200 μm, 20×, the magnified dotted 4 red boxes within ROI and the small square boxes of 120 × 120 µm on the right-side (for plaques and vessels) represents deposition characteristics on consecutive sections except the square box of 45 × 45 µm for the neuronal deposits). **b–c** Quantification of the number of plaques (*t*(6.997) = 2.378, *p = 0.0490) and percent plaque load (*t*(7.786) = 2.412, *p = 0.0432) stained with 4G8 antibody in the analyzed ROIs of TREM2 risk allele carriers (Plaque area cutoff = 10 µm^2^, n = 6 cases each for TREM2 variants and CV, unpaired t-test with Welch's correction). **d** Box and whiskers plot showing a significant increase in the plaque size of TREM2 variants compared to CV cases (n = 68,631 (CV) and n = 88,835 (TREM2 variants), ****p < 0.0001, Mann–Whitney test. Additional data are provided in Additional file 1: Figure S8b-c. **e**, **f** Quantification of the numbers of plaques (*t*(6.96) = 0.3754, ^ns^*p* = 0.7185) and the percent plaque load (*t*(4.366) = 0.5145, ^ns^*p* = 0.6319) stained with pSer8-Aβ antibody in the analyzed ROIs of TREM2 risk allele carriers (Plaque area cutoff = 30 µm^2^, n = 5 cases for TREM2 variants and n = 6 cases for CV, unpaired t-test with Welch's correction). Case#3, with highest fixation time, was omitted for the plaque count and size quantification as it was difficult to identify extracellular plaques stained with 1E4E11 (pSer8-Aβ) antibody. **g** Box and whiskers plot showing a significant decrease in the plaque size of TREM2 variants compared to CV cases (n = 17,456 (CV) and 13,291 (TREM2 variants), ****p < 0.0001, Mann–Whitney test. Additional data are provided in Additional file 1: Figure S8d-e. There was a significant increase of intracellular deposits of **h** nmAβ (*t*(9.138) = 2.429, *p = 0.0377) and **i** pSer8-Aβ (*t*(9.389) = 2.251, *p = 0.0497) in the analyzed cortical ROIs of TREM2 variant as compared to CV cases (n = 6 cases each for TREM2 variants and CV, unpaired t-test with Welch's correction). No data or numerical value was excluded unless otherwise stated. Each colored dot with respective gender symbol represents single case. Except for bot and whiskers plot, all data represent mean ± SEM

To assess the effect of rare disease associated TREM2 variants in human brain tissue, AD cases with TREM2^R62H/R62C^ variants and the clinical diagnosis for dementia were compared to cases with the TREM2 common variant (CV) also diagnosed with dementia. All cases fulfilled the criteria for neuropathological diagnosis of AD (Table 2). Sequential temporal neocortical sections were stained separately with the phosphorylation-state specific antibodies 7H3D6 (nmAβ), 1E4E11 (pSer8-Aβ), and with 4G8 that detects Aβ independently of the phosphorylation state. In addition to extracellular plaques, all cases showed Aβ depositions in the wall of cerebral blood vessels, and inside of neurons (Fig. 7a, Additional file 1: Figure S8a).

TREM2^R62H/R62C^ variant cases had increased number and size of 4G8 positive plaques resulting in a higher plaque load (% area) as compared to cases with the TREM2 common variant. Further, we also observed a higher number of smaller extracellular deposits stained with 4G8 antibody in TREM2 CV cases (Fig. 7a–d, Additional file 1: Figure S8b-c). Interestingly, only two of the six TREM2^R62H/R67C^ cases showed very few extracellular nmAβ positive deposits, while five out of six cases with the TREM2 CV showed abundant deposition of nmAβ (detected by antibody 7H3D6) in extracellular plaques, indicating that deposition of N-terminally non-modified Aβ species is reduced in cases with disease associated TREM2 variants. In contrast, pSer8-Aβ was present in extracellular plaques in TREM2^R62H/R62C^ and TREM2 CV cases, and prominently detected in the core of plaques. The number of pSer8-Aβ positive extracellular plaques and plaque load (% area) was not significantly different between TREM2^R62H/R62C^ and TREM2 CV cases (Fig. 7e-f). pSer8-Aβ deposits stained with 1E4E11 were overall smaller as deposits stained with antibody 4G8 that detects total Aβ with positively skewed plaque size distribution with both antibodies and in all cases (Fig. 7g, Additional file 1: Figure S8d-e). However, analysis of plaque size distribution revealed that the number and size of pSer8-Aβ positive extracellular deposits were decreased in TREM2^R62H/R62C^ cases as compared to TREM2 CV cases (Additional file 1: Figure S8d-e).

pSer8-Aβ and nmAβ was also detected in vessels in TREM2^R62H/R62C^ and TREM2 CV cases. However, further analyses on the regional and quantitative deposition of phosphorylated Aβ species in different disease stages would be required to assess a potential effect of TREM2 genotypes on CAA in mouse and human brains (Additional file 1: Figures S7e, S8a; Fig. 7a).

We also observed intraneuronal deposition of nmAβ and pSer8-Aβ species. Interestingly, the number of neurons with nmAβ and pSer8-Aβ positive intracellular deposits was significantly higher in TREM2^R62H/62C^ carriers as compared to TREM2 CV cases (Fig. 7h–i).

## Discussion

Here, we show that deletion of TREM2 or TREM2 disease associated variants lead to specific quantitative and qualitative changes of Aβ deposits in brains of APP transgenic mice and human cases with the diagnosis of  dementia, and that TREM2 not only modulates the composition of extracellular plaques, but also of intraneuronal deposits.

TREM2 plays a fundamental role in the regulation of microglial activity and the deposition of Aβ in extracellular plaques [28, 29, 31, 68]. Aβ exists in multiple variants with different lengths and post-translational modifications which differ in their aggregation behavior, biostability, deposition and neurotoxic properties [1, 42]. It was shown recently that phosphorylation of Aβ modulates the direct interaction with TREM2 and the internalization by microglia [31]. Phosphorylation at Ser8 also decreases the degradation of Aβ by the insulin degrading enzyme that can be secreted by microglia [39, 61].

To assess the role of TREM2 in the deposition of modified Aβ species in-vivo, we analyzed Aβ pathology in three different mouse models of AD. Consistent with previous reports [28, 76], TREM2 deletion was associated with increased Aβ deposition at younger (4–5 month) and older ages (12–15 month), and impaired microglial clustering around extracellular Aβ plaques [31]. Importantly, the phosphorylated Aβ species, pSer8-Aβ, selectively increased as compared to other Aβ species in brains of 5xFAD and APP/PS1L166P transgenic mice upon deletion of TREM2. Increased accumulation of phosphorylated Aβ species was also observed in brains of knock-in (KI) mice with the TREM2^T66M^ mutation that causes FTD-like syndrome and NHD in humans. In this regard, it is interesting to note that NHD cases could show AD characteristic neuropathological lesions, including senile plaques and neurofibrillary tangles [4]. Consistent with previous findings [31, 36, 40, 41], pSer8-Aβ is prominently detected in the core of extracellular plaques and intraneuronally. The deposition of pSer8-Aβ in both lesions was increased in brains of 12 M old TREM2^−/−^ as compared with TREM2^+/+^ in 5xFAD as well as APP/PS1L166P transgenic mice. Very similar alterations in the deposition of the different Aβ species were also observed in TREM2^T66M^ KI mice as compared to mice expressing functional TREM2, indicating that disease associated loss of function mutations also modulate the differential deposition of distinct Aβ variants.

Biochemical analysis of brain extracts revealed that TREM2 deficiency or the expression of the NHD associated T66M in APP transgenic mice increased the levels of pSer8-Aβ in form of oligomeric assemblies. Aβ oligomers exert neurotoxicity, and oligomer levels correlate with neuronal dysfunction in AD [3, 21, 50, 56, 57, 62, 80]. Aβ oligomers also seed fibrillization and promote the deposition in form of extracellular plaques [6, 10, 20, 32]. In addition, oligomers can be internalized by neurons resulting in Aβ accumulation within neurons [2, 9, 13, 56, 60]. Since TREM2 has been shown to selectively bind oligomeric assemblies of Aβ and promote microglial uptake [31, 44, 72, 82], the loss of TREM2 function could result in decreased clearance of oligomers and thereby lead to increased uptake by neurons, and deposition in extracellular plaques and the vasculature. Indeed, altered plaque development and CAA related pathology has recently been observed in mouse brain upon depletion of microglia [23, 59], showing that functional microglia are important to shape extracellular plaques and to restrict the deposition of Aβ in the brain vasculature. We also detected pSer8-Aβ and nmAβ in vessels of transgenic mouse brains, and it will be interesting the further investigate the role of TREM2 in the deposition and the potential pathophysiological implications of modified Aβ species in the vasculature.

The analysis of human cases with rare TREM2^R62H^ or TREM2^R62C^ variants showed significantly increased accumulation of phosphorylated Aβ species inside of neurons and alterations in plaque size, load and number, further indicating that impairment of TREM2 function mediates the formation and composition of AD characteristic lesions.

Thus, in addition to the previously described effects of TREM2 on plaque morphology, our data provide evidence for a critical role of TREM2 in restriction of intraneuronal Aβ deposits that are highly correlated with clinical presentation and disease progression [2, 7, 14, 15, 27, 63, 77]. Further, we show here that TREM2 also modulates the composition of these lesions. This is interesting, because changes in the composition of extracellular plaques and CAA, in particular the occurrence of phosphorylated Aβ, are associated with clinical manifestation and progression of AD [11, 46, 54, 64].

Although TREM2 loss of function mutations could also contribute to neurodegeneration independently of Aβ by impairment of brain energy metabolism [33], synapse dynamics [26, 48], and formation of neurofibrillary tangles [45], a recent study suggests an important role of Aβ pathology in the TREM2 dependent formation of tau pathology and brain atrophy [43]. Thus, the differential interaction of TREM2 with modified Aβ species might not only be important for the deposition and composition of different Aβ lesions, but also contribute to the development of tau pathology that together determine onset and progression of AD.

## Supplementary Information

**Additional file 1: Supplementary figures.**

**Additional file 2: Source data.**

## Data Availability

The data that support the findings of this study is available from the corresponding author upon reasonable request.

## Abbreviations

AD: Alzheimer’s disease
ADAM: A disintegrin and metalloproteinases
ApoE4: Apolipoprotein E4 allele
APP: Amyloid precursor protein
Aβ: Amyloid-β
CAA: Cerebral amyloid angiopathy
CV: Common variant
DAB: 3,3′-Diaminobenzidine
DAPI: 4′,6-Diamidino-2-phenylindole
DG: Dentate gyrus
EDTA: Ethylenediaminetetraacetic acid
EGTA: Ethylene glycol-bis(β-aminoethyl ether)-N,N,N′,N′-tetraacetic acid
EtOH: Ethanol
FAD: Familial Alzheimer's disease
FTD: Frontotemporal dementia
GWAS: Genome-wide association studies
IF: Immunofluorescence
IHC: Immunohistochemistry
IntDen: Integrated density
KI: Knock-In
NaDOC: Sodium Deoxycholate
N3pE-Aβ: N-terminal pyroglutamate-modified Amyloid-β
NaOH: Sodium hydroxide
NFTs: Neurofibrillary tangles
NHD: Nasu–Hakola disease
nmAβ: Non-modified Amyloid-β
PBS: Phosphate buffer saline
pSer8-Aβ: Phosphorylation at serine 8 of Amyloid-β
RIPA: Radioimmunoprecipitation assay
ROI: Region of interest
RSC: Retrosplenial cortex
RT: Room temperature
SDS: Sodium dodecyl sulphate
PAGE: Polyacrylamide gel electrophoresis
SSC: Somatosensory cortex
sTREM2: Soluble variants of Triggering receptor expressed on myeloid cells 2
TREM2: Triggering receptor expressed on myeloid cells 2
Tris: Tris(hydroxymethyl)aminomethane
TBS: Tris-buffered saline
WT: Wild type
