# Supplementary material for: TREM2 modulates differential deposition of modified and non-modified Aβ species in extracellular plaques and intraneuronal deposits
Source: Acta Neuropathol Commun. 2021 Oct 18;9:168. doi: 10.1186/s40478-021-01263-x (PMC8522217; doi:10.1186/s40478-021-01263-x)
Supplement: Supplementary file 1 — Additional file 1: Supplementary figures. [file 40478_2021_1263_MOESM1_ESM.pdf]

TREM2 modulates differential deposition of modified and non-modified Aβ species in extracellular plaques and intraneuronal deposits

Pranav Joshi[1], Florian Riffel[1], Sathish Kumar[1], Nàdia Villacampa[2,3], Sandra Theil[1], Samira Parhizkar[4], Christian Haass[4,5,6], Marco Colonna[7], Michael T. Heneka[2,3], Thomas Arzberger[6,8,9], Jochen Herms[5,6,8], Jochen Walter[1]*.

Additional file 1: Supplementary figures

Figure S1

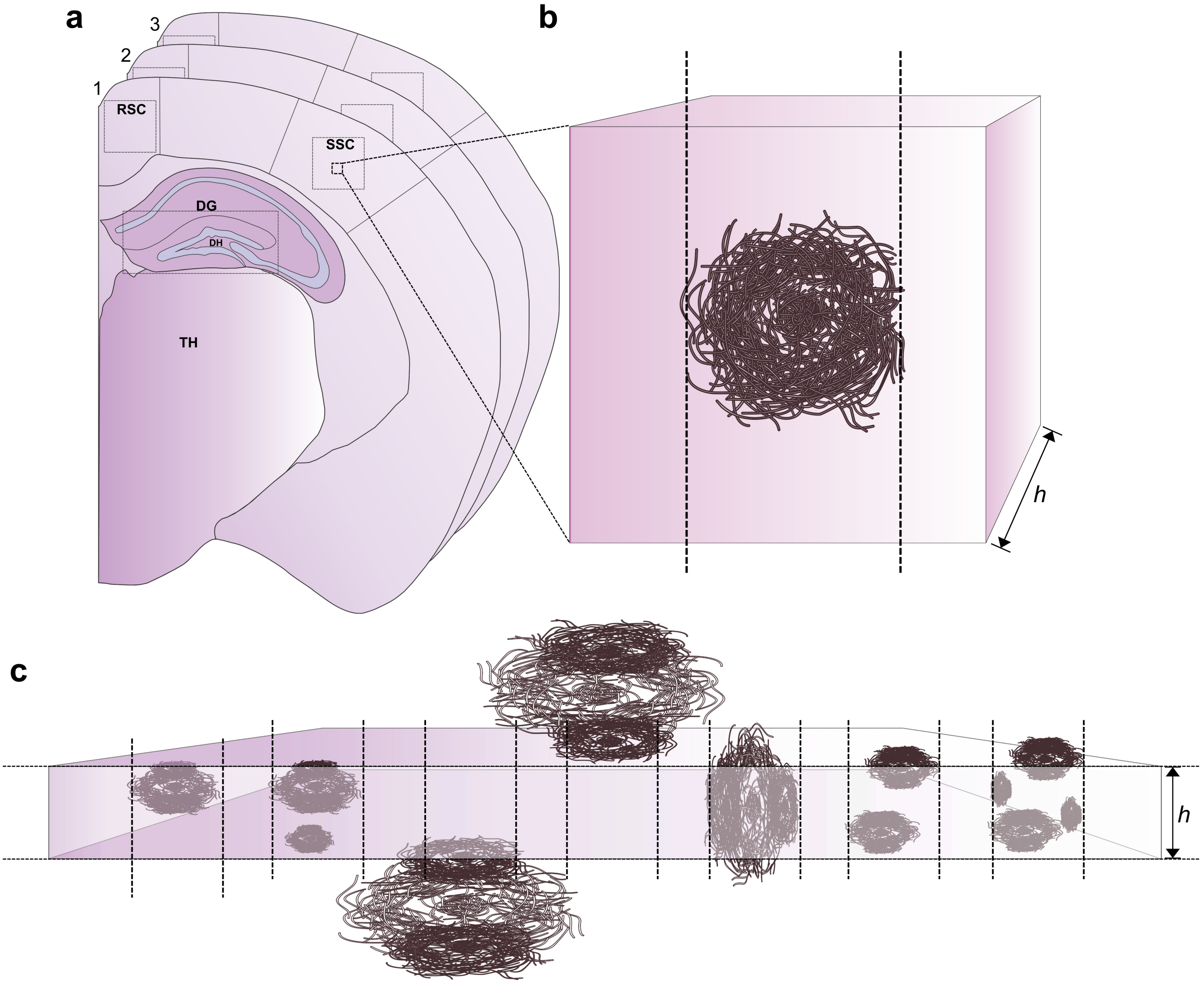

Figure S1| Visualization and approach of analysis of area and number of Aβ plaques.
(a) Showing different regions, RSC, SSC and DG regions in the coronal section of AD transgenic mouse brain analyzed in this study. For analysis of area and number of Aβ plaques three sections were used as depicted. **(b)** showing magnified region of interest (ROI) with Aβ plaque in 3D view with a perspective. **(c)** showing area and number of hypothetical Aβ plaque from **"b"** embedded in the brain section of thickness "*h*" μm at different visualizing plane. The Aβ plaque represented in **"b"** could be single, larger (outside the thickness), or multiple in the same region of interest hence the plaques and their quantified combined area in this study constitute the "lower boundary" of the plaque number volume density.

Figure S2

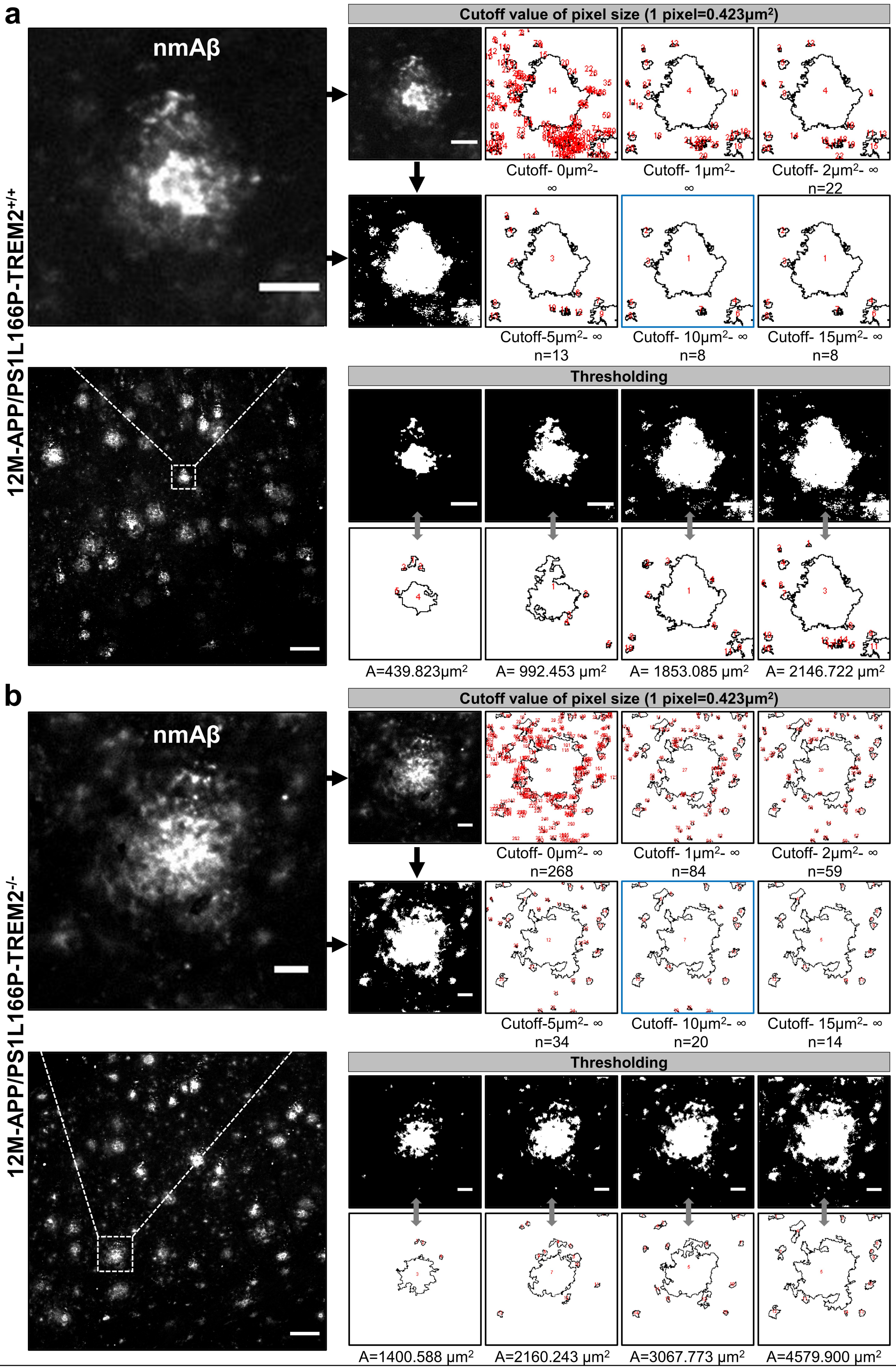

Figure S2| Analysis of area and size of Aβ plaque in the transgenic mouse brains.

(a-b) Representative images of SSC region of female 12M-APP/PS1L166P transgenic mouse brain with both TREM2 genotypes stained with nmAβ antibody. The cutoff value for the plaque size analysis determined the number of plaques hence default cutoff of 10μm^2 (blue box) was considered for the analysis. At this cutoff value, the number of plaques in TREM2$^{-/-}$ brains were significantly more as compared to TREM2$^{+/+}$ brains as showed in this study. Furthermore, the default thresholding also determined the area of plaques hence same thresholding was set to both groups and analysis were carried out. It is also important to note that, the area of plague was significantly more in TREM2$^{-/-}$ mouse brains as compared to TREM2$^{+/+}$ mouse brains even at the lowest threshold (scale bar=200μm and 20μm for the magnified images).

Figure S3

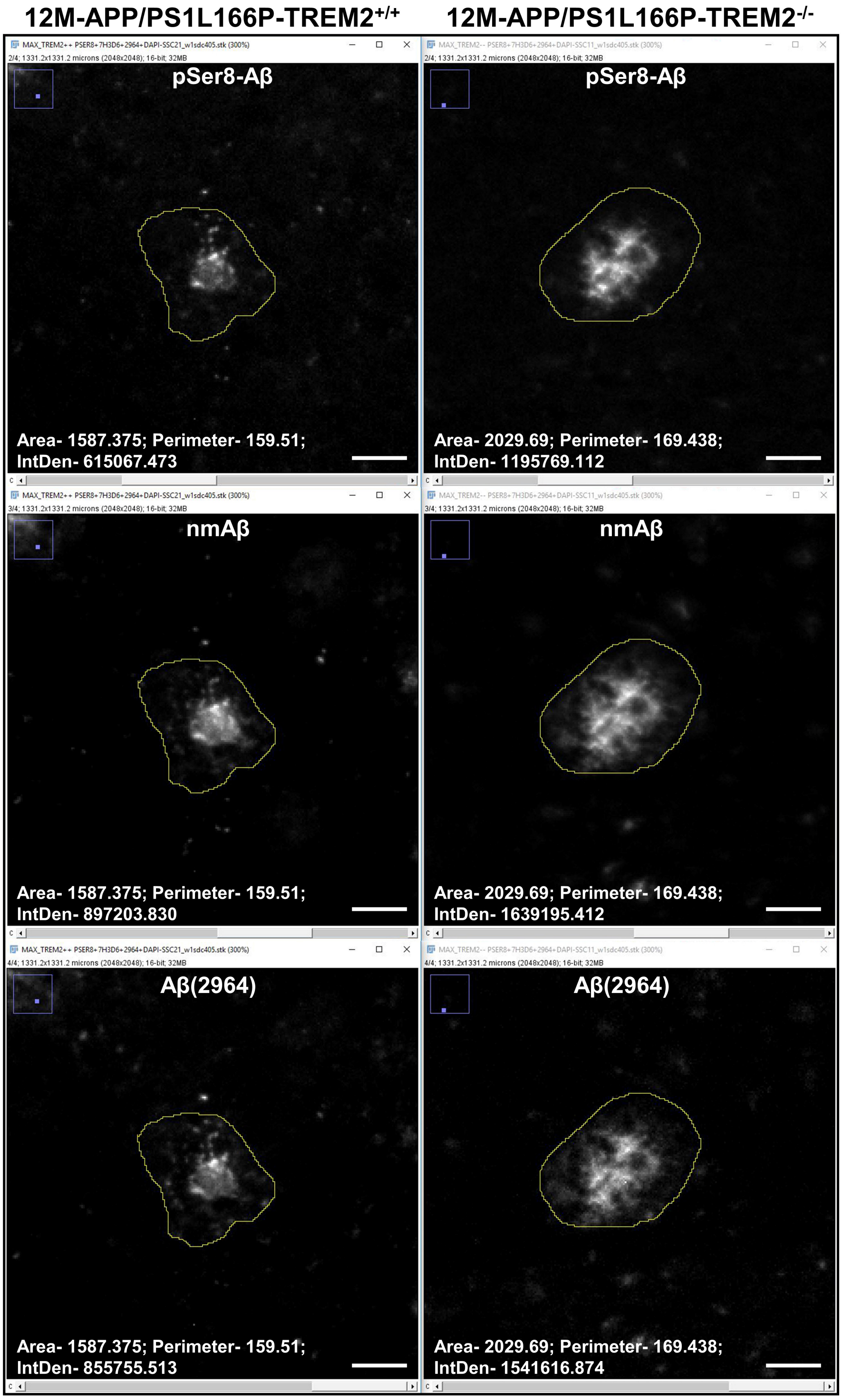

Figure S3| Analysis of staining intensity of Aβ plaque stained with different antibodies. Representative three confocal channels of SSC region of female 12M-APP/PS1L166P transgenic mouse brain with both TREM2 genotypes stained with three different Aβ antibodies. For the analysis of staining intensity ratio for the two different antibody stained plaques, a manual boundary surrounding the Aβ plaque (yellow color shown above) was made with “freehand selection” tool and the IntDen was measured keeping the same area for all three channels (scale bar=20µm).

Figure S4

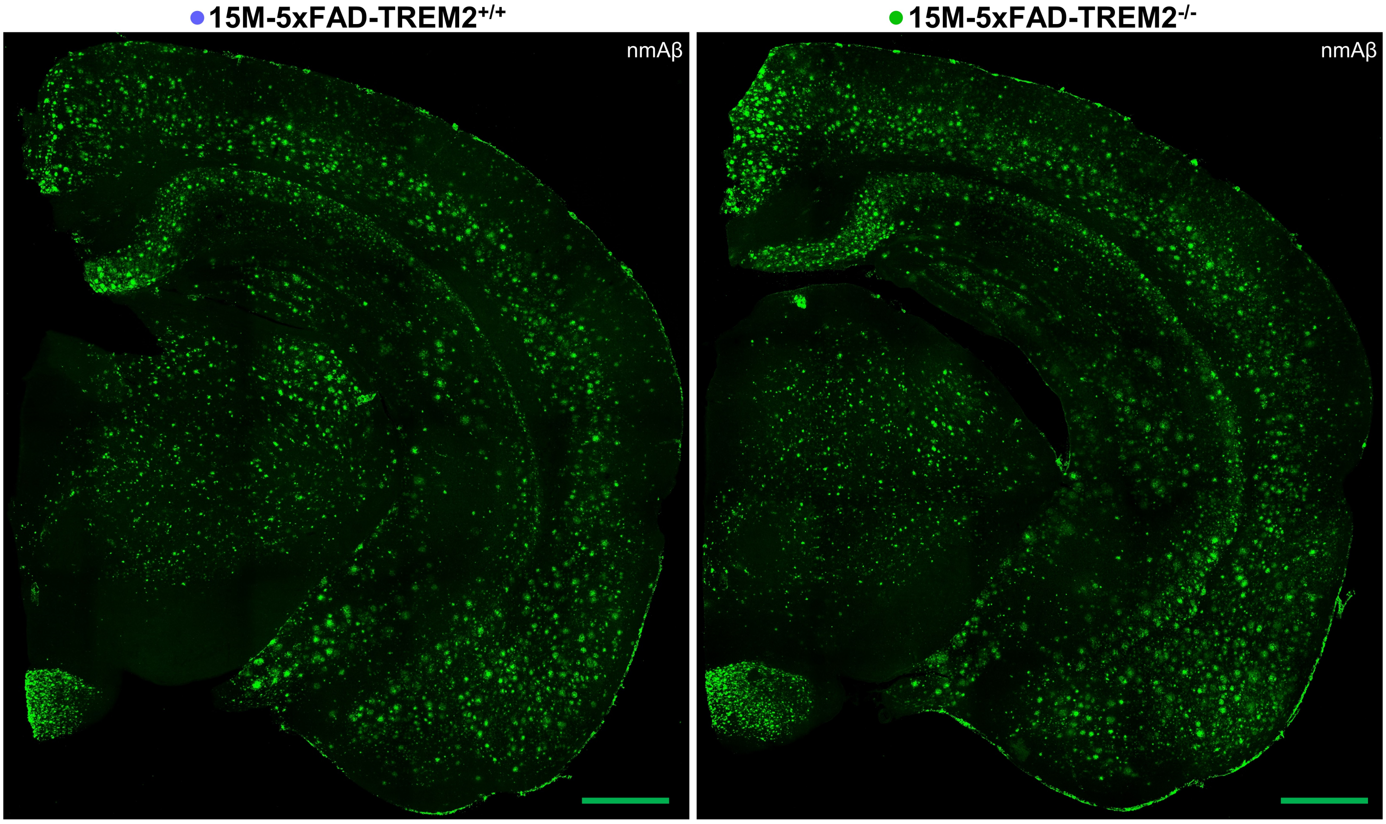

Figure S4| TREM2 deletion leads to increased deposition of Aβ in 5xFAD transgenic mouse brains. Representative nmAβ stained male 15M-5xFAD-TREM2$^{+/+}$ and TREM2$^{-/-}$ mouse brain sections (color scale bar=35mm,10x).

Figure S5

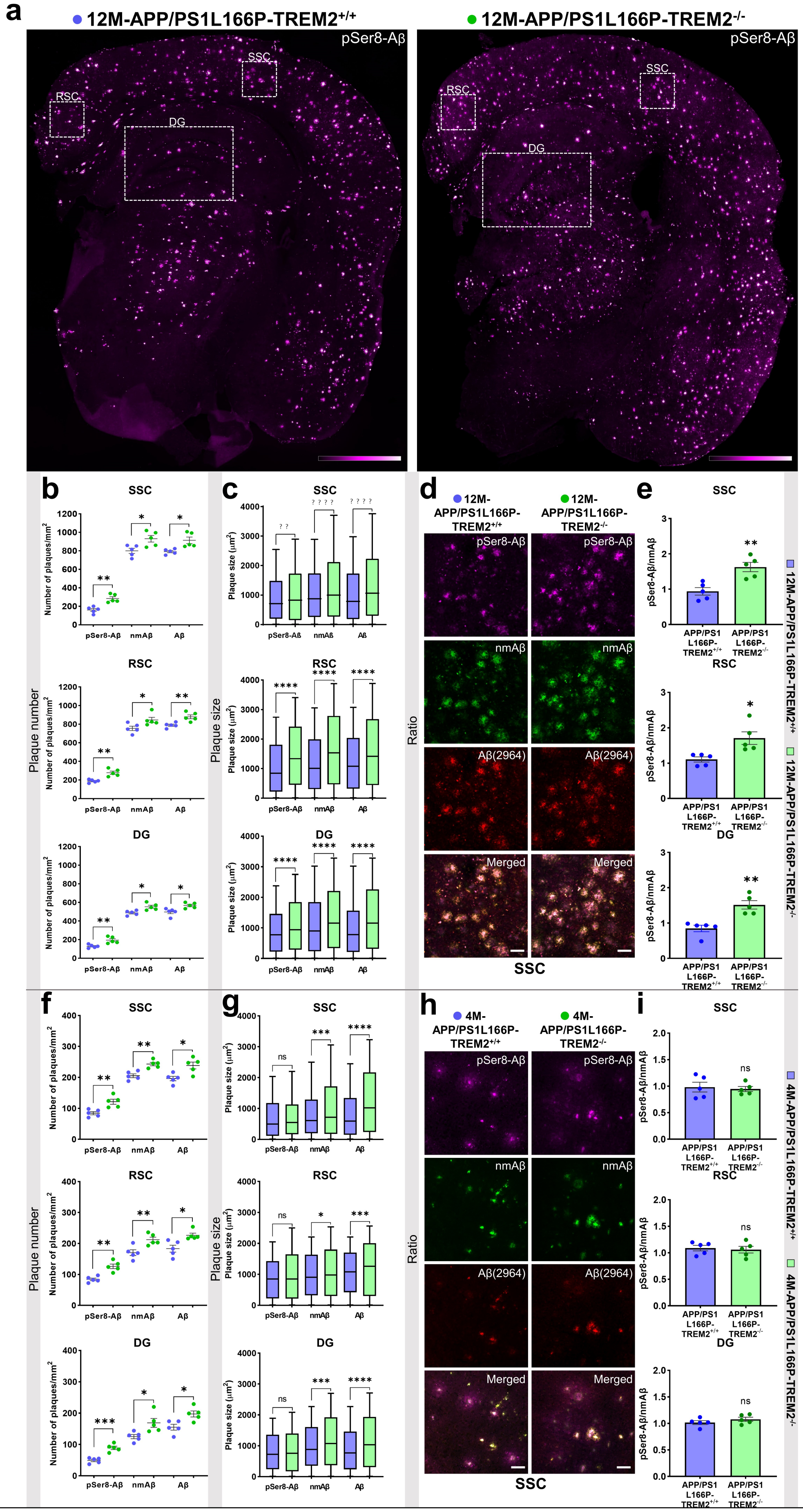

Figure S5| TREM2 deletion leads to increased deposition of pSer8-Aβ in APP/PS1L166P transgenic mouse brains.

(a) Representative pSer8-Aβ stained female 12M-APP/PS1L166P-TREM2$^{+/+}$ and TREM2$^{-/-}$ mouse brain sections-

(color scale bar=35mm, represents min/max pixel intensities, 10x). **(b)** Dot plots representing the increased number of plaques/mm^2, **(c)** Box and whiskers plots representing increased plaque size (μm^2) stained with pSer8-Aβ, nmAβ, and Aβ (2964) antibodies in the SSC, RSC, and DG of female 12M-APP/PS1L166P-TREM2$^{-/-}$ compared to TREM2$^{+/+}$ mice. **(d)** Representative images showing increased accumulation of pSer8-Aβ, nmAβ, and Aβ (2964) in SSC of female 12M-APP/PS1L166P-TREM2$^{-/-}$ compared to TREM2$^{+/+}$ mice (scale bar=50µm, 40xW). **(e)** Ratio of pSer8/nmAβ in SSC ($t(7.66)=4.053$,**$p=0.004$), RSC ($t(5.29)=3.19$,*$p=0.0241$), and DG ($t(7.48)=4.434$,**$p=0.0026$) of 12M-APP/PS1L166P-TREM2$^{-/-}$ compared with TREM2$^{+/+}$ mice. **(f)** Dot plots representing the increased number plaques/mm^2, **(g)** Box and whiskers plots representing increased plaque size (μm^2) stained with nmAβ and Aβ antibodies in the SSC, RSC, and DG of male 4M-APP/PS1L166P-TREM2$^{-/-}$ compared to TREM2$^{+/+}$ mice. **(h)** Representative images showing increased accumulation of pSer8-Aβ, nmAβ, and Aβ (2964) in SSC of male 4M-APP/PS1L166P-TREM2$^{-/-}$ compared to TREM2$^{+/+}$ mice (scale bar=50µm, 40xW). **(i)** Ratio of pSer8/nmAβ in SSC, RSC, and DG in male 4M-APP/PS1L166P-TREM2$^{-/-}$ compared with TREM2$^{+/+}$ mice. Each dot represents average value of number of plaques or ratio/animal. The box and whiskers plots represent min/max values of distribution of plaque size with the median (shown by the line dividing the box) and the dot plots represent mean ± SEM (n=5 animals, color- blue (APP/PS1L166P-TREM2$^{+/+}$) and green (APP/PS1L166P-TREM2$^{-/-}$), unpaired t-test with Welch's correction for analysis of the number and ratio while Mann-Whitney test for plaque size, $^{ns}p>0.05$, *$p< 0.05$, **$p<0.01$, ***$p<0.001$ or ****$p<0.0001$).

Figure S6

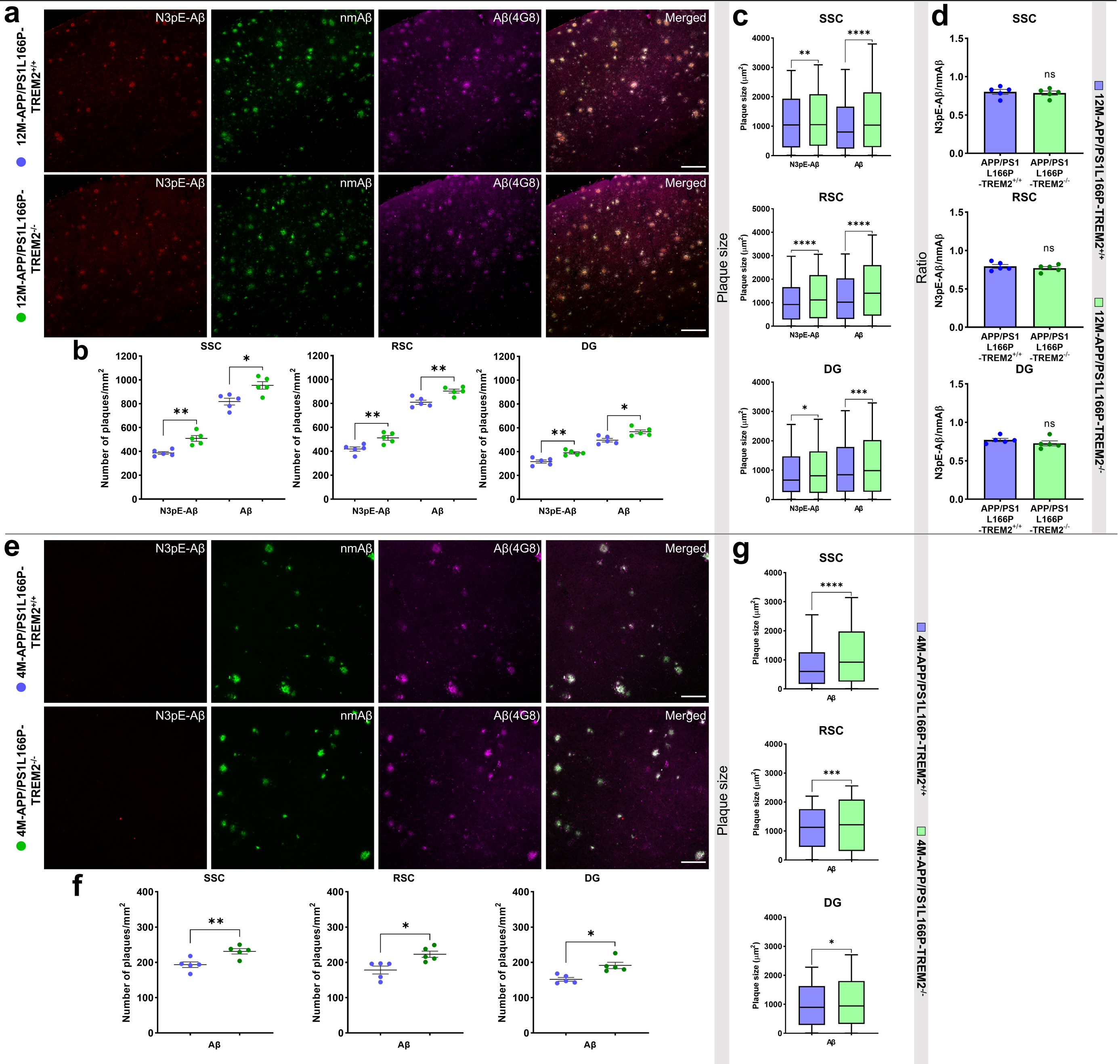

Figure S6| TREM2 deletion leads to increased deposition of N3pE-Aβ in APP/PS1L166P transgenic mouse brains.

(a) Representative images showing increased accumulation of N3pE-Aβ, and Aβ (4G8) in SSC of female 12M-APP/PS1L166P-TREM2$^{-/-}$ compared to TREM2$^{+/+}$ mice (scale bar=200μm,20x). **(b)** Dot plots representing the increased number of plaques/mm^2, **(c)** Box and whiskers plots representing increased plaque size (μm^2) stained with N3pE-Aβ, and Aβ (4G8) antibodies in the SSC, RSC, and DG of female 12M-APP/PS1L166P-TREM2$^{-/-}$ compared to TREM2$^{+/+}$ mice. **(d)** Ratio of N3pE-Aβ/nmAβ in the SSC, RSC, and DG of female 12M-APP/PS1L166P-TREM2$^{-/-}$ compared with TREM2$^{+/+}$ mice. **(e)** Representative images showing increased accumulation of Aβ (4G8) in SSC of male 4M-APP/PS1L166P-TREM2$^{-/-}$ compared to TREM2$^{+/+}$ mice (scale bar=200μm,20x). **(f)** Dot plots representing the increased number of plaques/mm^2, **(g)** Box and whiskers plots representing increased plaque size (μm^2) stained with Aβ(4G8) antibody in the SSC, RSC, and DG of male 4M-APP/PS1L166P-TREM2$^{-/-}$ compared to TREM2$^{+/+}$ mice. Each dot represents average value of number of plaques or ratio/animal. The box and whiskers plots represent min/max values of distribution of plaque size with the median (shown by the line dividing the box) and the dot plots represent mean ± SEM (n=5 animals, color- blue (APP/PS1L166P-TREM2$^{+/+}$) and green (APP/PS1L166P-TREM2$^{-/-}$), unpaired t-test with Welch's correction for analysis of the number and ratio while Mann-Whitney test for plaque size, $^{ns}p>0.05$, $*p<0.05$, $**p<0.01$, $***p<0.001$ or $****p<0.0001$).

Figure S7

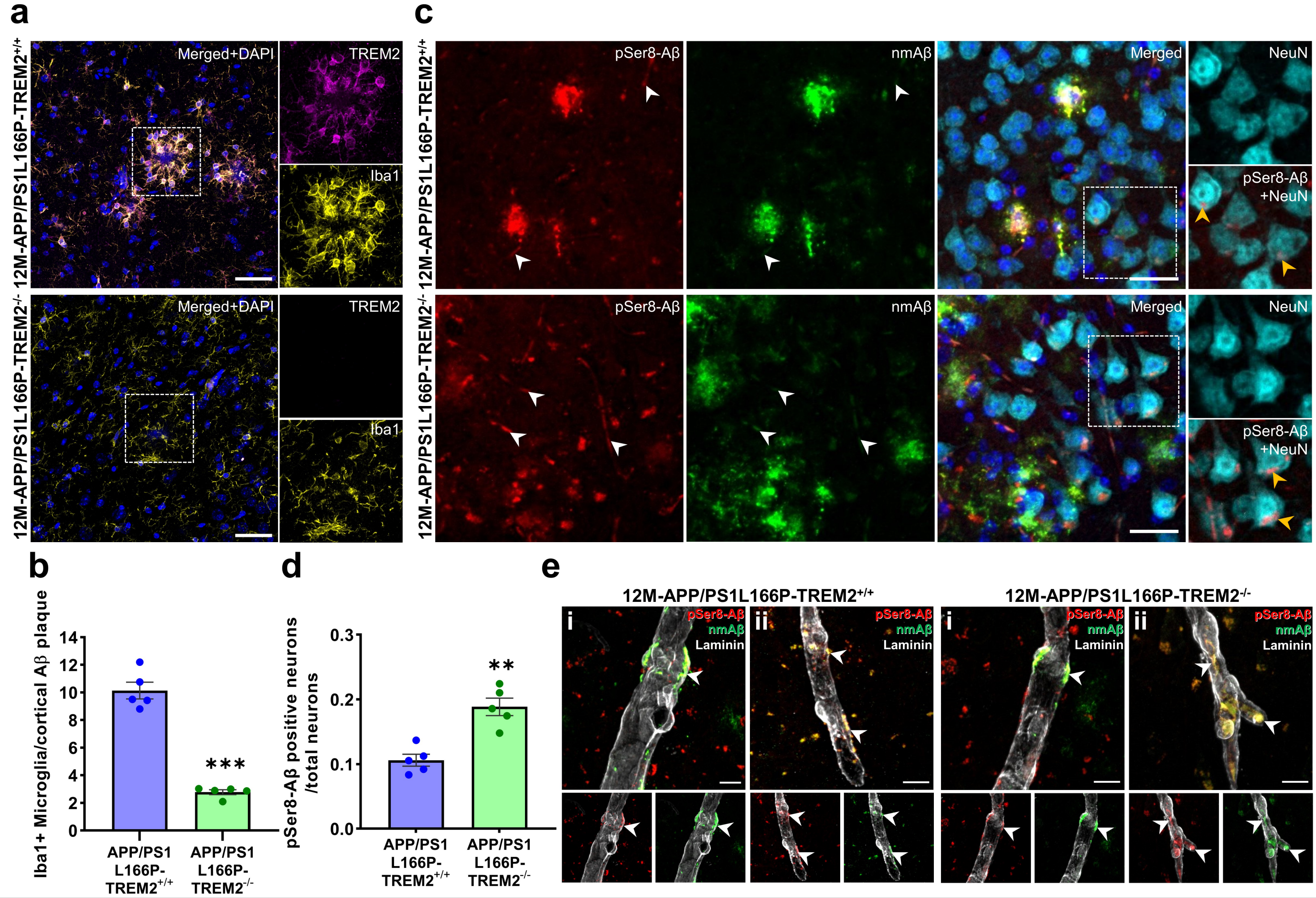

Figure S7| Loss of microglial barrier functions upon TREM2 deletion leads to increased intraneuronal pSer8-Aβ deposits in the APP/PS1L166P transgenic mouse brains.

(a) Representative IF images of TREM2 along with microglial marker, Iba1, in female 12M-APP/PS1L166P-TREM2$^{+/+}$ and TREM2$^{-/-}$ mice. Dotted white boxes indicate the area shown at higher magnification. (scale bar=50µm, 20x). **(b)** Quantification showing decreased Iba1^{+ve} plaque associated microglia surrounding cortical plaques in the female 12M-APP/PS1L166P-TREM2$^{-/-}$ compared with TREM2$^{+/+}$ mice (t(4.67)=11.68,***p=0.0001). Each dot represents the mean value of microglia count surrounding 30 cortical plaques/animal. **(c)** Representative IF images of pSer8-Aβ and nmAβ localized in extracellular plaques, vessels (white arrowheads) and within NeuN positive neurons (yellow arrowheads) in SSC of female 12M-APP/PS1L166P-TREM2$^{+/+}$ and TREM2$^{-/-}$ mice. Dotted white boxes indicate the area shown at higher magnification (scale bar=50µm, 40xW).**(d)** Quantification of intraneuronal pSer8-Aβ normalized to total number of neurons showed significantly increased neuronal pSer8-Aβ deposits in the SSC of female 12M-APP/PS1L166P-TREM2$^{-/-}$ as compared to TREM2$^{+/+}$ mice (t(7.078)=5.06, **p=0.0014). **(e)** Representative cortical artery (i) and capillary (ii) stained with Laminin showing pSer8-Aβ and nmAβ deposits in the SSC of female 12M-APP/PS1L166P-TREM2$^{+/+}$ and TREM2$^{-/-}$ mice (indicated with arrow) (Scale bar=10µm, 63xW). All data represent mean ± SEM (n=5 animals, color- blue (APP/PS1L166P-TREM2$^{+/+}$) and green (APP/PS1L166P-TREM2$^{-/-}$), unpaired t-test with Welch's correction).

Figure S8

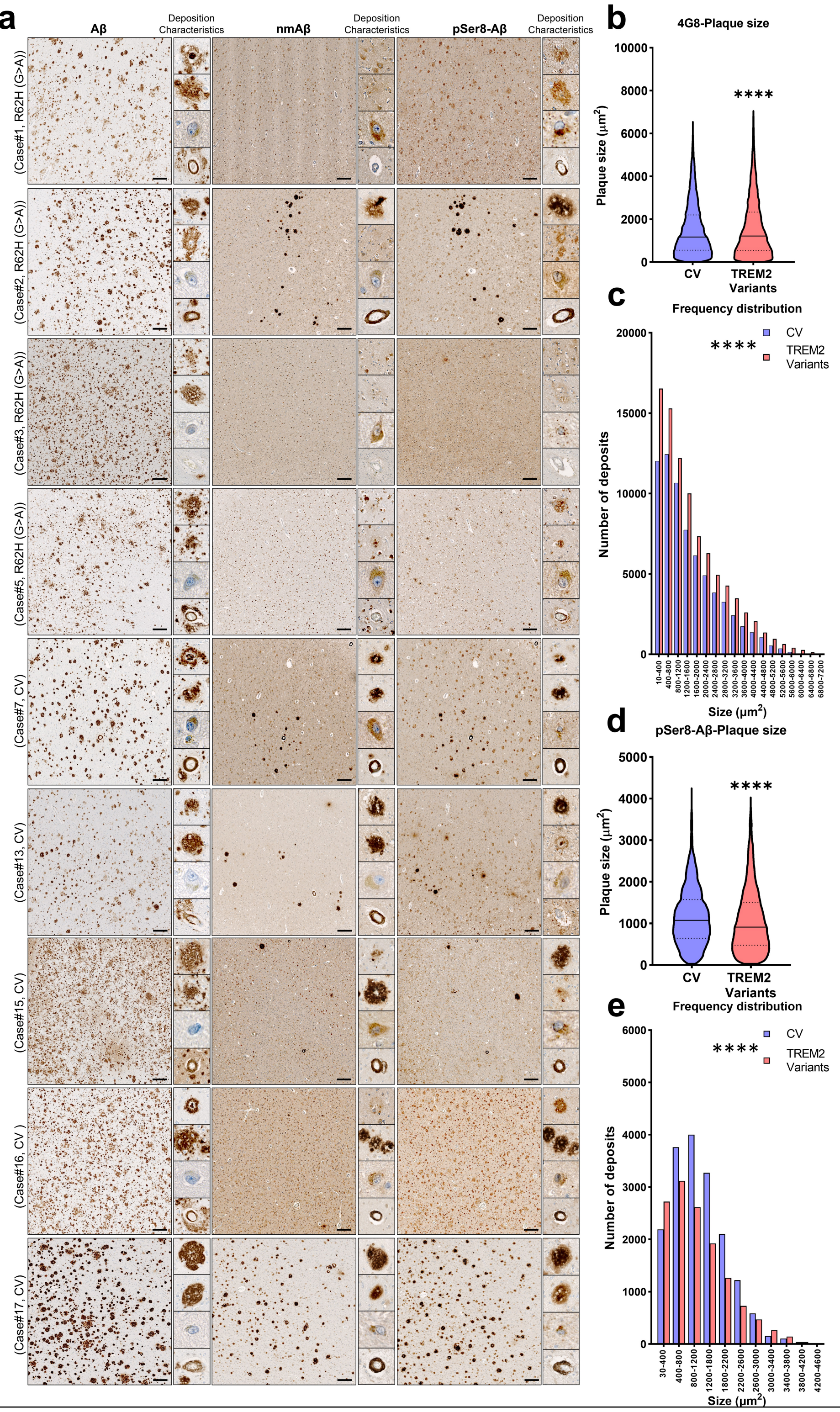

Figure S8| TREM2 ectodomain mutation leads to differential deposition of post-translationally modified Aβ species in the human brains.
(a) Temporal neocortex of human AD patients with and without the indicated TREM2 variants stained for Aβ, nmAβ, and pSer8-Aβ by IHC (scale bar=200µm, 20x). Each small square box at the right side are either 120×120-140×140µm (for plaques/vessels) or 45×45µm for neurons **(b)** Violin plot represents 4G8 stained plaque size (in µm^2) distribution and **(c)** frequency distribution histogram with total counted 4G8 stained plaques in analyzed cortical ROIs/group. (Plaque area cutoff=10µm^2, n=68631 (CV) and n=88835 (TREM2 variants)).**(d)** Violin plot represents plaque size distribution (in µm^2) and **(e)** frequency distribution histogram with total counted pSer8-Aβ stained plaques in analyzed cortical ROIs/group. (Plaque area cutoff=30µm^2, n=17456 (CV) and 13291 (TREM2 variants)). Case#3, with highest fixation time, was omitted for the plaque count and size quantification as it was difficult to identify extracellular plaques stained with 1E4E11(pSer8-Aβ) antibody. Comparison of two groups of violin plot were done by Mann-Whitney test and the frequency distribution of all values by Kolmogorov-Smirnov test wherein ****p<0.0001.

Table S1

Sr. No.	Antibody	Source/Catalog number	Species	Dilution
**Primary antibodies**				
1	Aβ- 4G8	Sigma-Aldrich Cat# A1349, RRID:AB_476683	Mouse monoclonal	IF-1:250; IHC-1:4000; ICC-250; WB-1:1000
2	Aβ- 2964	In-house made (Genscript)	Rabbit polyclonal	IF-1:250; WB-1:1000
3	Non-modified Aβ (nmAβ)-7H3D6	In-house made (Genscript)	Rat monoclonal	IF-1:250; IHC-1:100; WB-1:250
4	pSer8-Aβ-1E4E11	In-house made (Genscript)	Mouse monoclonal	IF-1:250; IHC-1:50; WB-1:250
5	N3pE-Aβ	Immuno-Biological Laboratories Cat# JP18591, RRID:AB_1630801	Rabbit polyclonal	IF-1:100
6	Laminin	Sigma-Aldrich Cat# L9393, RRID:AB_477163	Rabbit polyclonal	IF-1:25
7	TREM2-AF1729	R and D Systems Cat# AF1729, RRID:AB_354956	Sheep polyclonal	IF-1:50
8	Iba1	Synaptic Systems Cat# 234 003, RRID:AB_10641962	Rabbit polyclonal	IF-1:1000; ICC-1:500
		Millipore Cat# MABN92, RRID:AB_10917271	Mouse monoclonal	IF-1:300
9	NeuN	Abcam Cat# ab104225, RRID:AB_10711153	Rabbit polyclonal	IF-1:500
10	β-actin	Sigma-Aldrich Cat# A1978, RRID:AB_476692	Mouse monoclonal	WB-1:5000
**Secondary antibodies**				
1	Alexa Fluor 488	Thermo Fisher Scientific Cat# A-11055, RRID:AB_2534102	Donkey anti-goat	IF-1:500; ICC-1:1000
2	Alexa Fluor 488	Thermo Fisher Scientific Cat# A-21202, RRID:AB_141607	Donkey anti-mouse	IF-1:500
3	Alexa Fluor 488	Thermo Fisher Scientific Cat# A-11029, RRID:AB_2534088	Goat anti-mouse	IF-1:500; ICC-1:1000
4	Alexa Fluor 488	Thermo Fisher Scientific Cat# A-21208, RRID:AB_2535794	Donkey anti-rat	IF-1:500
5	Alexa Fluor 488	Thermo Fisher Scientific Cat# A-11034, RRID:AB_2576217	Goat anti-rabbit	IF-1:500; ICC-1:1000
6	Alexa Fluor 546	Thermo Fisher Scientific Cat# A-21098, RRID:AB_2535752	Donkey anti-sheep	IF-1:500
7	Alexa Fluor 546	Thermo Fisher Scientific Cat# A-10040, RRID:AB_2534016	Donkey anti-rabbit	IF-1:500; ICC-1:1000
8	Alexa Fluor 546	Thermo Fisher Scientific Cat# A-10036, RRID:AB_2534012	Donkey anti-mouse	IF-1:500; ICC-1:1000
9	Alexa Fluor 546	Thermo Fisher Scientific Cat# A-11035, RRID:AB_2534093	Goat anti-rabbit	IF-1:500; ICC-1:1000
10	Alexa Fluor 546	Thermo Fisher Scientific Cat# A10040, RRID:AB_2534016	Donkey anti-rabbit	IF-1:500; ICC-1:1000
11	Alexa Fluor 546	Thermo Fisher Scientific Cat# A-11081, RRID:AB_2534125	Goat anti-rat	IF-1:500
12	Alexa Fluor 647	Thermo Fisher Scientific Cat# A-21236, RRID:AB_2535805	Goat anti-mouse	IF-1:500; ICC-1:1000
13	Alexa Fluor 647	Thermo Fisher Scientific Cat# A-21245, RRID:AB_2535813	Goat anti-rabbit	IF-1:500; ICC-1:1000
14	Mouse HRP	Sigma-Aldrich Cat# A9044, RRID:AB_258431	Mouse polyclonal	WB-1:15000
15	IRDye® 680CW	LI-COR Biosciences Cat# 926-68072, RRID:AB_10953628	Donkey anti-mouse	WB-1:5000
		LI-COR Biosciences Cat# 926-68076, RRID:AB_10956590	Goat anti-rat	
		LI-COR Biosciences Cat# 926-68073, RRID:AB_10954442	Donkey anti-Rabbit	
16	IRDye® 800CW	LI-COR Biosciences Cat# 926-32212, RRID:AB_621847	Donkey anti-mouse	WB-1:5000
		LI-COR Biosciences Cat# 926-32219, RRID:AB_1850025	Goat anti-rat	
		LI-COR Biosciences Cat# 926-32213, RRID:AB_621848	Donkey anti-Rabbit	

Table S1| List of primary and secondary antibodies used in this study. IF-immunofluorescence, ICC-Immunocytochemistry, WB-Western blotting, HRP- Horseradish peroxidase.
