# Supplementary material for: TREM2 modulates differential deposition of modified and non-modified Aβ species in extracellular plaques and intraneuronal deposits
Source: Acta Neuropathol Commun. 2021 Oct 18;9:168. doi: 10.1186/s40478-021-01263-x (PMC8522217; doi:10.1186/s40478-021-01263-x)
Supplement: Supplementary file 2 — Additional file 2: Source data. [file 40478_2021_1263_MOESM2_ESM.pdf]

TREM2 modulates differential deposition of modified and non-modified Aβ species in extracellular plaques and intraneuronal deposits

Pranav Joshi[1], Florian Riffel[1], Sathish Kumar[1], Nàdia Villacampa[2,3], Sandra Theil[1], Samira Parhizkar[4], Christian Haass[4,5,6], Marco Colonna[7], Michael T. Heneka[2,3], Thomas Arzberger[6,8,9], Jochen Herms[5,6,8], Jochen Walter[1]*.

Additional file 2: Source data

Source data 1

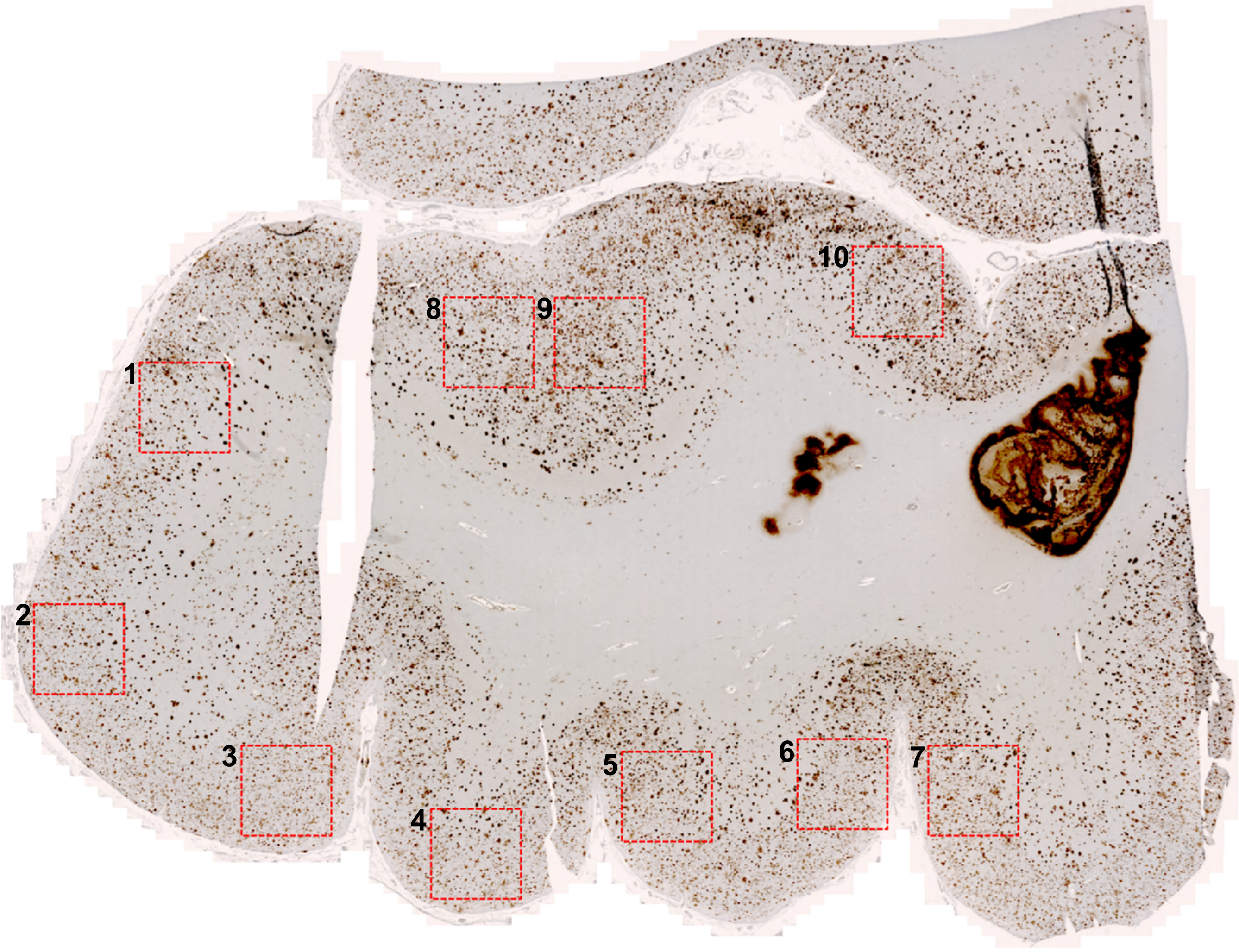

Source data 1| Selection of region of interest (ROI) from the human brain sections for the analysis. Showing representative randomly selected 10 ROIs each of 2×2mm from the human brain section (Case#1) stained with 4G8 antibody. Please refer the methods section for further details.

Source data 2

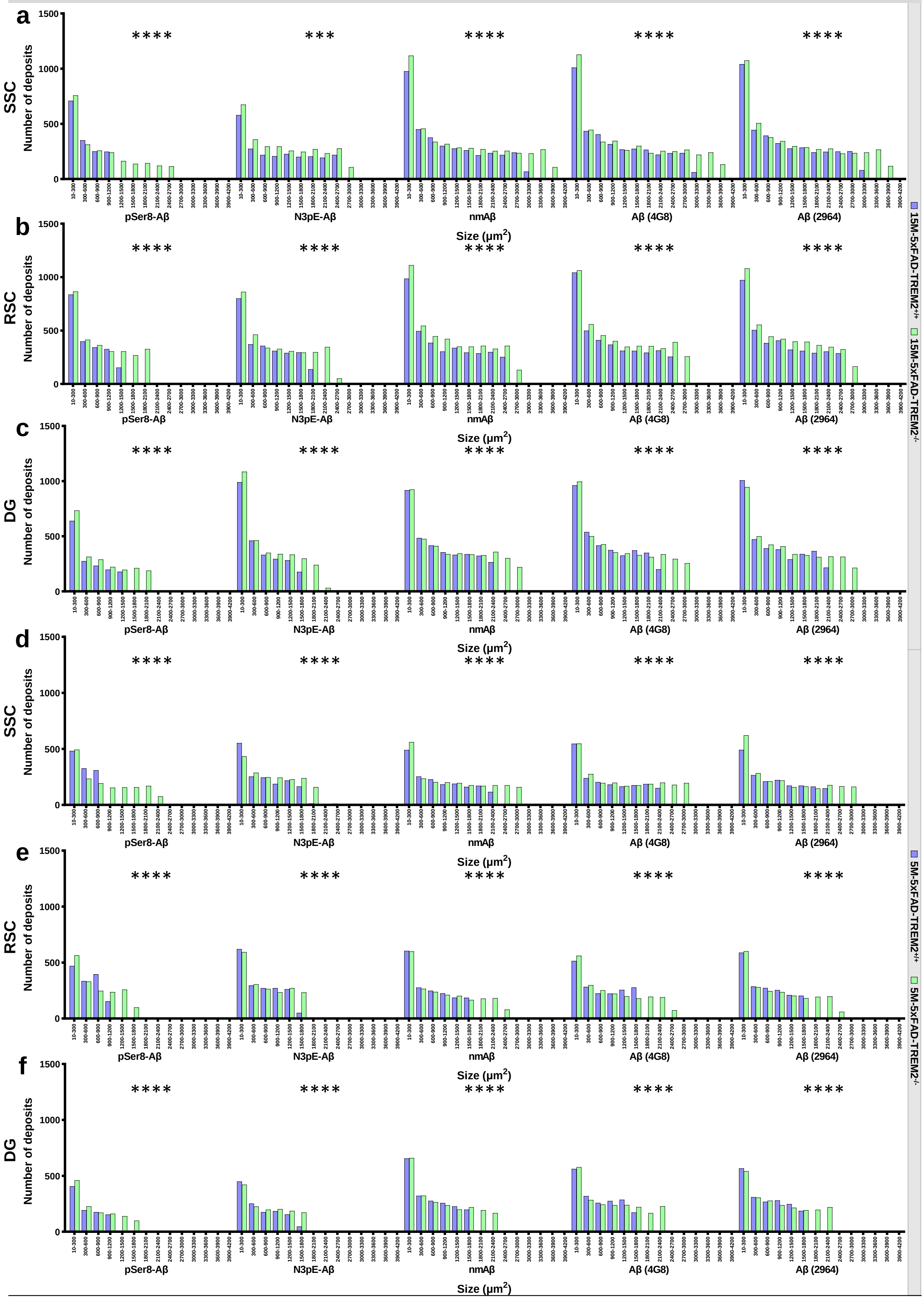

Source data 2| Size distribution of Aβ plaques stained with different Aβ antibodies in 5xFAD-TREM2$^{+/+}$ and 5xFAD-TREM2$^{-/-}$ mice.

Frequency distribution histogram representing plaque size (µm^2) distribution of total counted Aβ plaques stained with antibodies detecting modified and non-modified forms of Aβ in SSC, RSC and DG regions of **(a-c)** 15M old male and **(d-f)** 5M old male mice. Comparison of two frequency distributions with total number of deposits was done by Kolmogorov-Smirnov test. (n= 5 animals, size of all individual plaques counted/group (refer Table 1), color-blue (5xFAD-TREM2$^{+/+}$) and green (5xFAD-TREM2$^{-/-}$), ***p<0.001 or ****p<0.0001).

Source data 3

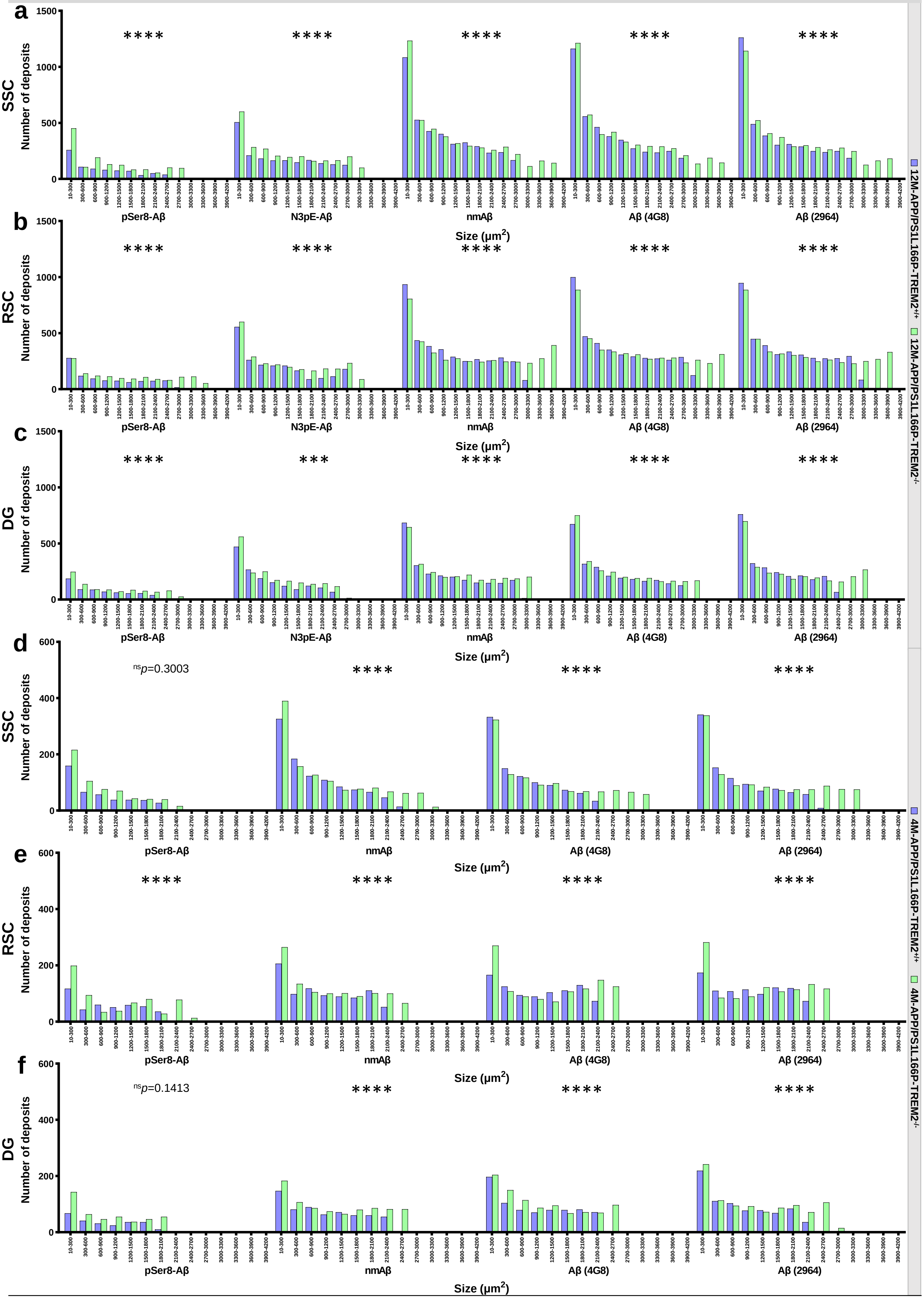

Source data 3| Size distribution of Aβ plaques stained with different Aβ antibodies in APP/PS1L166P-TREM2$^{+/+}$ and TREM2$^{-/-}$ mice.

Frequency distribution histogram representing plaque size (µm^2) distribution of total counted Aβ plaques stained with antibodies detecting modified and non-modified forms of Aβ in SSC, RSC and DG regions of **(a-c)** 12M old female and **(d-f)** 4M old male mice. Comparison of two frequency distributions with total number of deposits was done by Kolmogorov-Smirnov test. (n=5 animals, size of all individual plaques counted/group (refer Table 1), color-blue (APP/PS1L166P-TREM2$^{+/+}$) and green (APP/PS1L166P-TREM2$^{-/-}$), $^{ns}p>0.05$, $^{***}p<0.001$ or $^{****}p<0.0001$).

Source data 4

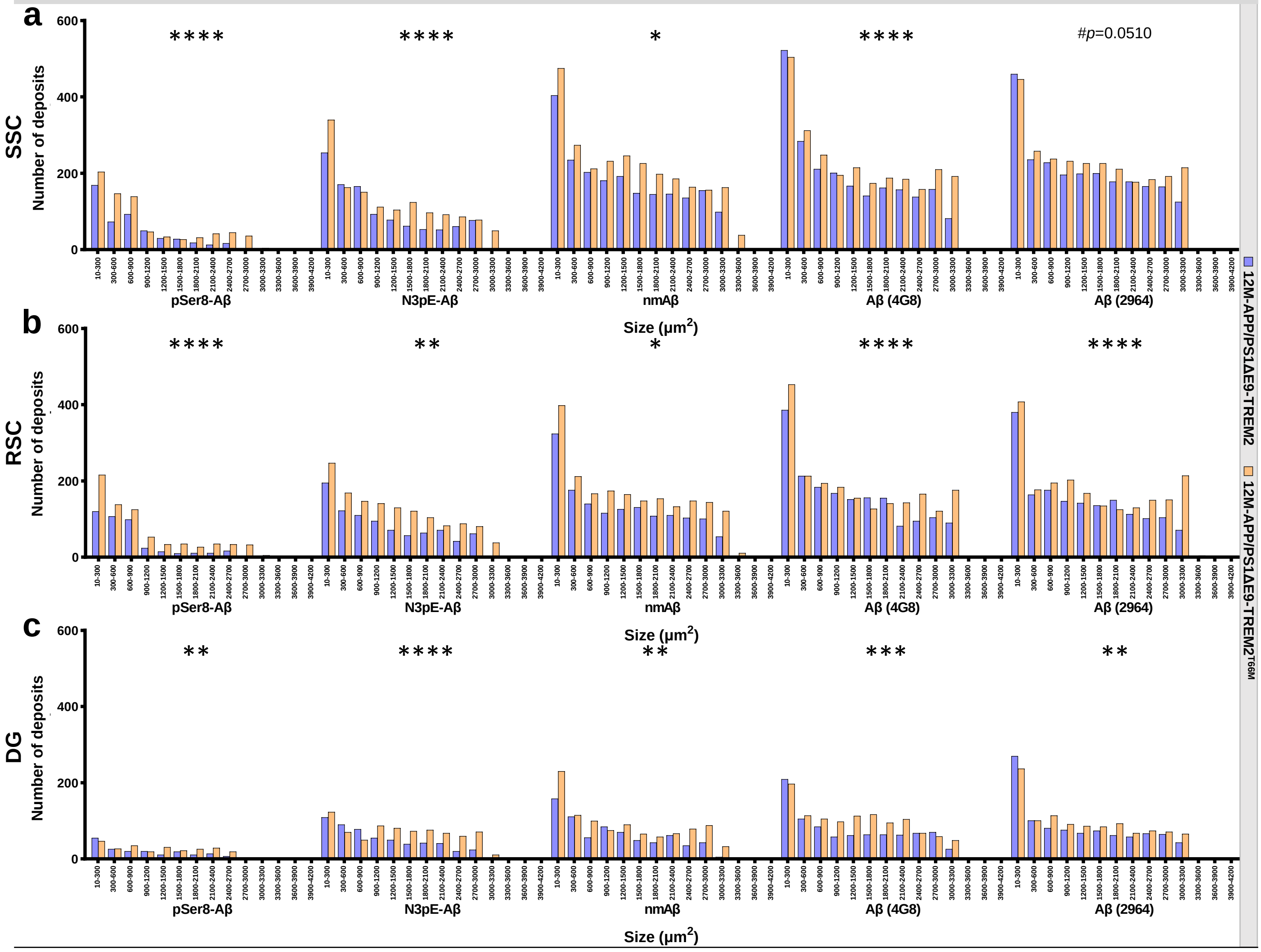

Source data 4| Size distribution of Aβ plaques stained with different Aβ antibodies in APP/PS1ΔE9-TREM2 and APP/PS1ΔE9-TREM2^{T66M} mice.

Frequency distribution histogram representing plaque size (µm^2) distribution of total counted Aβ plaques stained with antibodies detecting modified and non-modified forms of Aβ in SSC, RSC and DG regions of **(a-c)** 12M old female mice. Comparison of two frequency distributions with total number of deposits was done by Kolmogorov-Smirnov test. (n=3 animals, size of all individual plaques counted/group (refer Table 1), color- blue (APP/PS1ΔE9-TREM2) and orange (APP/PS1ΔE9-TREM2^{T66M}), $^{\#}p$>0.05, *p< 0.05, **p<0.01, ***p<0.001 or ****p<0.0001).

Source data 5

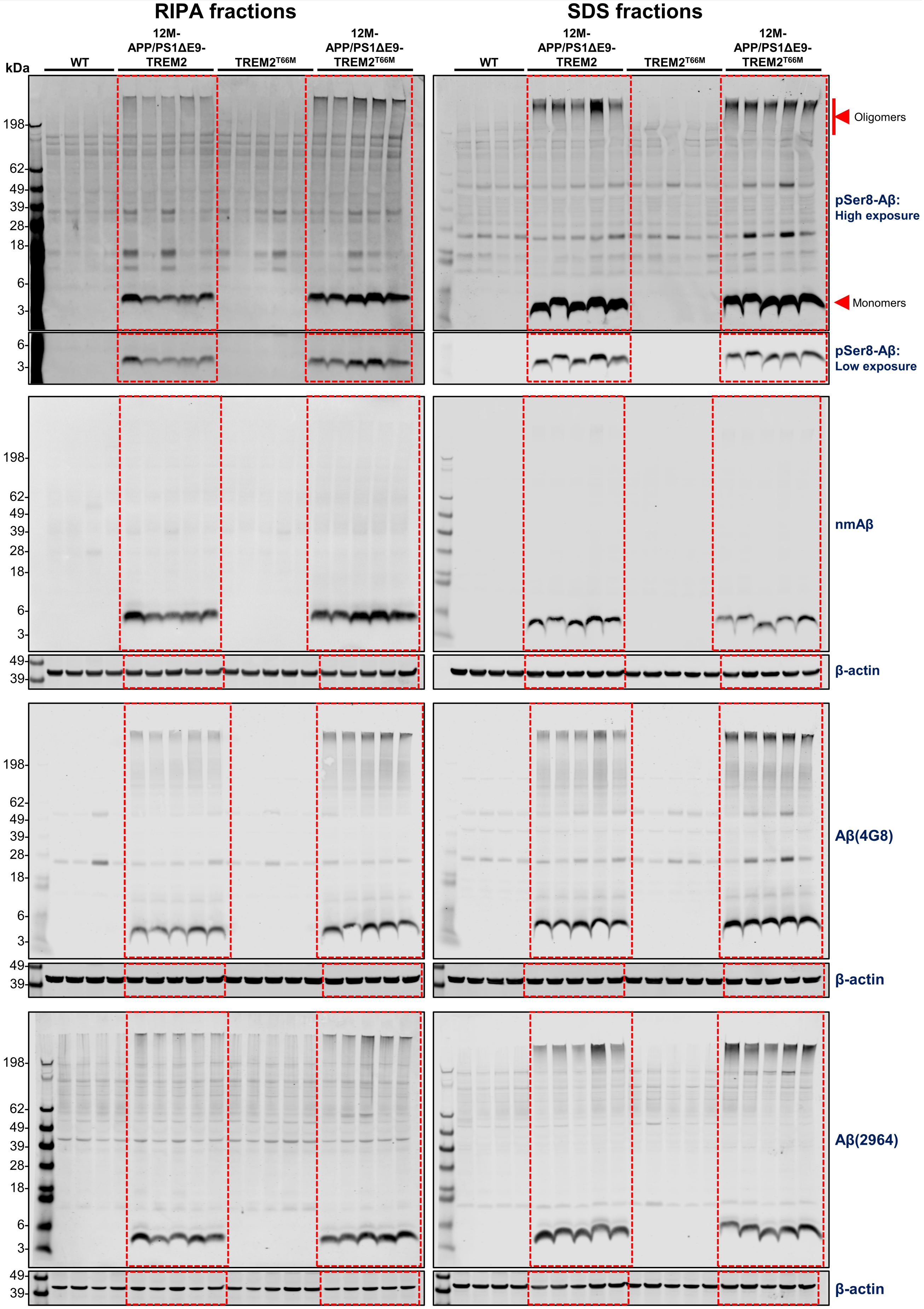

Source data 5| Original immunoblots stained with antibodies detecting modified and non-modified forms of Aβ. The dotted line boxes were cropped to represent in the main Figure 6.
